# A case for glycerol as an acceptable additive for single-particle cryoEM samples

**DOI:** 10.1107/S2059798321012110

**Published:** 2022-01-01

**Authors:** Benjamin Basanta, Marscha M. Hirschi, Danielle A. Grotjahn, Gabriel C. Lander

**Affiliations:** a Department of Integrative Structural and Computational Biology, Scripps Research, La Jolla, CA 92037, USA

**Keywords:** cryoEM, glycerol, sample buffer, apoferritin, aldolase

## Abstract

Buffer-composition and sample-preparation guidelines for cryo-electron microscopy are geared towards maximizing imaging contrast and reducing electron-beam-induced motion. These pursuits often involve the minimization or the complete removal of additives that are commonly used to facilitate proper protein folding and minimize aggregation. Among these admonished additives is glycerol, a widely used osmolyte that aids protein stability. In this work, it is shown that the inclusion of glycerol does not preclude high-resolution structure determination by cryoEM, as demonstrated by an ∼2.3 Å resolution reconstruction of mouse apoferritin (∼500 kDa) and an ∼3.3 Å resolution reconstruction of rabbit muscle aldolase (∼160 kDa) in the presence of 20%(*v*/*v*) glycerol. While it was found that generating thin ice that is amenable to high-resolution imaging requires long blot times, the addition of glycerol did not result in increased beam-induced motion or an inability to pick particles. Overall, these findings indicate that glycerol should not be discounted as a cryoEM sample-buffer additive, particularly for large, fragile complexes that are prone to disassembly or aggregation upon its removal.

## Abstract 2

It is shown that the inclusion of glycerol in single-particle cryoEM buffers does not preclude high-resolution structure determination, as demonstrated by an ∼2.3 Å resolution reconstruction of mouse apoferritin (∼500 kDa) and an ∼3.3 Å resolution reconstruction of rabbit muscle aldolase (∼160 kDa) in the presence of 20%(*v*/*v*) glycerol.

## 1. Introduction

The initial, and often the most challenging, task associated with detailed structural characterization of a biological target is the biochemical purification of the macromolecule, and this remains a significant bottleneck in the field of structural biology. Often, substantial efforts are made in optimizing the recombinant expression or reconstitution of the targeted macromolecule and the subsequent isolation of the target into a solution that optimally preserves its structural composition and stability. Glycerol is a linear, three-carbon polyol that is widely used in buffer solutions to enhance macromolecular stability, since it prevents protein unfolding by stabilizing aggregation-prone intermediates and favoring more compact protein conformations (Vagenende *et al.*, 2009 ▸). Many purified complexes strictly require the presence of glycerol for biochemical stability and are susceptible to unfolding or aggregation in its absence. Despite the notable benefits for target stability, the inclusion of glycerol in the final purification buffer has been strongly discouraged by the cryo-electron microscopy (cryoEM) community.

There are three main arguments that have been made against the inclusion of glycerol in cryoEM buffers. Firstly, the inclusion of glycerol in the buffer increases the solvent density, which is thought to decrease contrast to the detriment of data quality and structure solution. The density of a 20% glycerol solution at −200°C is 1.033 g cm^−3^, while the average density of protein is 1.181 g cm^−3^. Given that our ability to resolve macromolecular structures embedded in vitrified buffer depends on the differential scattering of electrons of the target complex versus the surrounding buffer, it is thought that increasing the density of the buffer will decrease the attainable image contrast. Secondly, glycerol has previously been shown to significantly increase beam-induced motion during image acquisition. For instance, the addition of 50% glycerol increased the motion of gold fiducials by up to tenfold (Karuppasamy *et al.*, 2011 ▸). Motion of the sample during acquisition will blur high-resolution features, and even motion-correction methodologies cannot recover high-resolution information from images containing substantial beam-induced motion. Thirdly, it is widely believed that the addition of glycerol to the vitrification buffer increases the sensitivity to radiation damage, as ‘bubbling’ is often observed in EM samples containing glycerol (Frederik *et al.*, 1991 ▸; Karuppasamy *et al.*, 2011 ▸). This bubbling is thought to be caused by the production of hydrogen gas upon the irradiation of biological materials and small organic molecules such as glycerol. Thus, the presence of glycerol in the buffer may cause widespread bubbling and encumber structure determination.

These arguments against the use of glycerol in sample preparation for cryoEM structure determination resulted in the widespread avoidance of glycerol, even when macromolecular complexes require the biochemical stabilization that it provides. However, the limited literature on the subject describes experiments involving a glycerol concentration of 50%(*v*/*v*) (Karuppasamy *et al.*, 2011 ▸), which is higher than those typically considered for aiding solubility during protein purification [5–40%(*v*/*v*); Bondos & Bicknell, 2003 ▸], imaging thick, dense biological samples that bear little resemblance to those prepared for single-particle analysis (Frederik *et al.*, 1991 ▸) and using relatively low imaging voltages (100–120 keV), which are known to cause greater radiation damage than the current voltages typically used for single-particle cryoEM (Peet *et al.*, 2019 ▸). In order to expand the utility of cryoEM imaging for samples that require glycerol for stability, we tested the effect of a buffer containing 20% glycerol on our ability to solve high-resolution reconstructions of two model specimens, apoferritin and aldolase, using current imaging methodologies. The chosen glycerol concentration is the midpoint between the maximum generally considered for protein stabilization during purification [40%(*v*/*v*)] and the concentration deemed practical for size-exclusion chromatography [<10%(*v*/*v*)], which is a ubiquitous step in protein purification. As expected, the increased viscosity of the buffer made optimal grid preparation more challenging and the associated increase in ice thickness complicated image analysis. We were nonetheless able to determine high-resolution structures of both model specimens. These findings demonstrate that the inclusion of glycerol in the final cryoEM buffer of a targeted sample does not abolish the ability to determine a high-resolution structure, thereby widening the range of biological targets that can be studied by cryoEM.

## 2. Materials and methods

### 2.1. Sample preparation

Mouse apoferritin (heavy chain) was prepared as described previously (Wu *et al.*, 2020 ▸). The protein was expressed in *Escherichia coli* BL21(DE3)pLys cells from a pET-24a vector (Danev *et al.*, 2019 ▸). The cells were grown to an OD_600_ of 0.5 at 37°C in 500 ml LB medium with shaking at 220 rev min^−1^. Isopropyl β-d-1-thiogalactopyranoside was then added to 1 m*M* to induce expression. Growth continued under the same conditions for 3 h, after which the cells were harvested and pelleted at 4000*g* for 10 min at 4°C. The pellet was resuspended in 20 ml lysis buffer (30 m*M* HEPES pH 7.5, 300 m*M* NaCl, 1 m*M* MgSO_4_) supplemented with 1 mg ml^−1^ lysozyme and cOmplete Protease Inhibitor Cocktail (Roche). The cells were lysed by sonication and the resulting lysate was clarified by centrifugation at 20 000*g* for 30 min at 4°C. The clarified lysate was heated for 10 min at 70°C to precipitate endogenous *E. coli* proteins. Denatured proteins were pelleted by spinning at 20 000*g* for 15 min at 4°C. Ammonium sulfate was added to the clarified lysate to a concentration of 60%(*w*/*v*), followed by gentle stirring on ice for 10 min. The precipitate was centrifuged at 14 000*g* for 20 min. The resulting pellet was resuspended in 2 ml cold phosphate-buffered saline and dialyzed against Q1 buffer (30 m*M* HEPES pH 7.5, 1 m*M* DTT, 20 m*M* NaCl). After dialysis, the protein was further diluted by adding an equal volume of Q1 buffer and loaded onto a HiTrap Q HP anion-exchange chromatography column (GE Healthcare) equilibrated in Q1 buffer. The column was washed with four volumes of Q1 buffer and eluted with a 0–100% gradient of SEC buffer (30 m*M* HEPES pH 7.5, 1 m*M* DTT, 500 m*M* NaCl) over three column volumes. Apoferritin eluted between 150 and 200 m*M* NaCl, which was confirmed by SDS–PAGE. The relevant fractions were pooled and concentrated to 10–20 mg ml^−1^ for loading onto a Superdex 200 Increase 10/300 (GE Healthcare) size-exclusion chromatography column equilibrated with 30 m*M* HEPES pH 7.5, 150 m*M* NaCl, 1 m*M* DTT. The peak fractions corresponding to apoferritin were pooled and concentrated to 15 mg ml^−1^. For long-term storage, glycerol was added to 5%(*v*/*v*).

Rabbit muscle aldolase was prepared as described previously (Herzik *et al.*, 2017 ▸). Lyophilized powder was purchased from Sigma–Aldrich and solubilized in 20 m*M* HEPES pH 7.5, 50 m*M* NaCl to a final concentration of around 3 mg ml^−1^. To remove aggregated protein, the protein solution was filtered through a 0.2 µm filter and loaded onto a Sepharose 6 10/300 (GE Healthcare) column equilibrated in solubilization buffer for size-exclusion chromatography. The fractions containing native, pure rabbit muscle aldolase were pooled and concentrated to 14.5 mg ml^−1^.

### 2.2. Cryo-electron microscopy sample handling and grid preparation

Prior to application onto cryoEM grids, the apoferritin sample was diluted to 5 mg ml^−1^ using either SEC buffer (final glycerol concentration of 1.7%; ‘no added glycerol’ experiments) or SEC buffer supplemented with glycerol (‘20% glycerol’ experiments) such that the final glycerol concentration was 20%(*v*/*v*). 4 µl of apoferritin sample was applied onto UltrAuFoil R1.2/1.3 300 mesh grids (Quantifoil Micro Tools GmbH) that had been freshly plasma-cleaned for 7 s at 15 W (75% nitrogen/25% oxygen atmosphere) using a Solarus plasma cleaner (Gatan). The grids were manually blotted using Whatman No. 1 filter paper and immediately plunge-frozen into liquid ethane cooled by liquid nitrogen using a custom-built manual plunger located in a cold room (≥95% relative humidity, 4°C). For the samples with 1.7% glycerol we blotted for 6 s. Expecting high glycerol concentrations to pose issues with ice thickness, based on anecdotal evidence, we prepared grids of samples with 20% glycerol by blotting for 6, 8 and 10 s (one grid per blotting time). We chose manual blotting for two reasons. (i) By placing the blotting paper parallel to the grid, we tend to obtain squares of similar quality across the whole grid surface. Consequently, when optimal conditions are found, a greater surface of the grid is likely to display the optimal ice conditions, in contrast to the gradient obtained by blotting using a Vitrobot (Iancu *et al.*, 2006 ▸). (ii) We have previously observed substantially more thinning of the ice in the center of the holes when preparing grids with the Vitrobot Mark IV (Thermo Fisher Scientific) than when preparing grids by manual blotting, and we attribute this difference to the lag period between blotting and plunging when using the Vitrobot. This thinning is sufficient to exclude particles from the hole center where images are taken.

We screened the abovementioned grids under cryogenic conditions on an FEI F20 electron microscope operating at 200 keV at a nominal magnification of 62 000× (a pixel size of 1.774 Å on a Tietz 4k × 4k CCD camera) with a defocus of −1.6 µm using the *Leginon* automated electron-microscopy package (Suloway *et al.*, 2005 ▸) for image acquisition. We began screening by locating squares with the largest area covered in electron-transparent ice and acquiring images from 2–3 holes in their center. We then explored the rest of the grid, keeping notes on the number of similar-looking squares. Once other squares with similar ice coverage had been located, we acquired additional images to verify that acceptable ice could reliably be identified. Grids where most squares appeared to be covered with large areas of thin ice, and images acquired from them presented thin ice with good particle contrast, were deemed to be optimal. Based on the CCD images, the ice seemed to be of sufficient quality and thickness for high-resolution imaging using a 10 s blot for samples containing glycerol and 6 s for samples without additional glycerol.

Prior to application onto cryoEM grids, the aldolase stock was diluted to 1.6 mg ml^−1^ using buffer supplemented with glycerol such that the final glycerol concentration was 20%(*v*/*v*). As for the apoferritin sample, 4 µl of aldolase sample was applied onto UltrAuFoil R1.2/1.3 300 mesh grids (Quantifoil Micro Tools GmbH) that had been freshly plasma-cleaned for 6 s at 15 W (75% nitrogen/25% oxygen atmosphere) using a Solarus plasma cleaner (Gatan). The grids were manually blotted using Whatman No. 1 filter paper and immediately plunge-frozen into liquid ethane cooled by liquid nitrogen using a custom-built manual plunger located in a cold room (≥95% relative humidity, 4°C). In order to optimize the ice thickness in the presence of glycerol, we prepared grids by blotting for 6, 8, 10 and 12 s, with two grids prepared at each blotting time. As performed for the apoferritin samples, we screened these grids in search of thin ice and and to assess the particle concentrations in each hole on an F20 with a CCD. We verified that the ice thickness decreased as the blot time increased, except for the 12 s blotting, where surprisingly thick ice became apparent at the center of the squares. Based on these observations, we decided to collect data on grids blotted for 10 s. For the data-collection session in the Talos Arctica using a direct electron detector we prepared eight new grids, trying to accommodate for any variability in the manual blotting process: one grid at 7 s blotting, three grids at 8 s, one grid at 9 s and two grids at 10 s. Data were acquired on a grid that had been blotted for 10 s, which had the thinnest ice.

For single-particle data collection and ice measurement at 300 keV for apoferritin samples, new grids were prepared using the same grid-preparation parameters as for single data acquisition at 200 keV. Data for these samples were acquired on the first grid that presented acceptable ice. Four additional grids were prepared for this session at room temperature and ambient humidity (45%), but they did not present improvements over those prepared at 4°C and 95% humidity. For single-particle data collection and ice measurement at 300 keV for aldolase samples, four new grids were prepared: one at 7 s blotting, two at 8 s blotting and one at 9 s blotting. Consistent with the results from imaging at 200 keV, the 9 s blotted grid presented the best ice conditions.

### 2.3. CryoEM data acquisition for single-particle analysis

For the grids imaged at 200 keV, movies of frozen-hydrated aldolase and apoferritin in the presence of 20% glycerol were collected using a Talos Arctica TEM (Thermo Fisher Scientific) with a field emission gun operating at 200 keV equipped with a K2 Summit direct electron detector (Gatan). Alignments were performed as described previously to attain parallel illumination (Herzik *et al.*, 2017 ▸), and image shift was used to image holes in a 4 × 4 array for apoferritin and a 2 × 2 array for aldolase, using stage movement only to shift between subsequent arrays. Movies were collected in counting mode (1.15 and 0.91 Å per pixel, respectively) at doses of 50 and 80 e^−^ Å^−2^, respectively (110 frames of 100 ms and 96 frames of 200 ms, respectively) and a nominal defocus of −1.0 and −1.3 µm, respectively. Movies of apoferritin in the absence of glycerol were collected under the same imaging conditions as apoferritin in the presence of 20% glycerol, with the only difference being the collection of 113 frames of 100 ms.

For the grids imaged at 300 keV, movies of frozen-hydrated apoferritin and aldolase in the presence of 20% glycerol were collected using a Titan Krios TEM (Thermo Fisher) with a field emission gun operating at 300 keV equipped with a K2 Summit direct electron detector (Gatan). Image shift was used to image holes in a 4 × 4 array for apoferritin and a center-of-five array for aldolase, using stage movement only to shift between subsequent arrays. Movies were collected in counting mode (1.026 and 1.045 Å per pixel, respectively) at a dose of 50 e^−^ Å^−2^ (65 frames of 100 ms and 50 frames of 200 ms, respectively) and defocus ranges of between −0.5 and −1.5 µm for apoferritin and between −1.25 and −1.75 µm for aldolase. Tomography data were acquired on a small region of these same samples prior to data collection for single-particle analysis (see Section 6 for details).

All cryoEM data for single-particle analysis were acquired using the *Leginon* automated data-collection software (Suloway *et al.*, 2005 ▸) and were pre-processed in real time using the *Appion* package (Lander *et al.*, 2009 ▸). Pre-processing consisted of beam-induced motion correction and contrast-transfer function (CTF) estimation. Frame alignment and dose weighting were performed using *MotionCor*2 (Zheng *et al.*, 2017 ▸) without binning with 5 × 5 patches, seven iterations, a global *B* factor of 500 Å^2^ and a local *B* factor of 100 Å^2^. CTF estimation was performed using *CTFFIND*4 (Rohou & Grigorieff, 2015 ▸) on the aligned but non-dose-weighted frames using the following parameters: field size 1024, amplitude contrast 0.07, minimum resolution 50 Å, maximum resolution 4 Å. Real-time CTF estimation was only used to monitor data quality during collection.

### 2.4. Analysis of single-particle data

#### 2.4.1. Apoferritin without added glycerol at 200 keV

We used *RELION* 3.1 (Zivanov *et al.*, 2018 ▸) for data analysis of apoferritin without added glycerol (Supplementary Fig. S1). We aligned dose-weighted movie frames using *MotionCor*2 from within *RELION* 3.1 in order to enable subsequent Bayesian polishing, and estimated the initial CTF parameters using *CTFFIND*4 from within *RELION* 3.1. For particle picking, we began by manually picking ∼50 particles from 19 randomly selected micrographs. We extracted the picked particles in boxes of 192 pixels, without binning (1.15 Å per pixel), and classified them into five 2D classes. We selected the one class that most clearly resembled apoferritin as a template for template-based picking in the *RELION* 3.1 *AutoPick* node. This resulted in 876 503 picks from 1707 micrographs. 2D classification (five classes, Tau = 2, 25 iterations) of these picks produced three classes with clear secondary-structure features, which contained 875 694 particles. EMD entry EMD-21024 was low-pass filtered to 30 Å to serve as the initial model for auto-refinement (*O* symmetry imposed) of these particles, which yielded a reconstruction at 3.9 Å resolution using an FSC cutoff of 0.143. Particles were grouped by image shift followed by per-micrograph defocus and astigmatism refinement, combined with beam-tilt refinement, yielding a reconstruction at 2.4 Å resolution. Bayesian polishing, followed by 3D classification without alignment (*O* symmetry imposed, four classes, Tau = 20, 100 iterations) and further CTF refinement, increased the resolution to 2.3 Å (FSC = 0.143).

#### 2.4.2. Apoferritin in 20%(*v*/*v*) glycerol at 200 keV

We used *RELION* 3.1 for data analysis of apoferritin in the presence of 20% glycerol (Supplementary Fig. S2). We aligned dose-weighted movie frames using *MotionCor*2 from within *RELION* 3.1 in order to enable subsequent Bayesian polishing, and estimated the initial CTF parameters using *CTFFIND*4 from within *RELION* 3.1. We initially manually picked 2261 particles from 20 randomly selected micrographs. We extracted the picked particles in boxes of 192 pixels, without binning (1.15 Å per pixel), and classified them into five 2D classes. We used the four classes that most clearly resembled apoferritin as templates for template-based picking in the *RELION* 3.1 *AutoPick* node. This resulted in 830 042 picks from 2254 micrographs. 2D classification (five classes, Tau = 2, 25 iterations) of these picks produced two classes with clear secondary-structure features, which contained 829 923 particles. EMD entry EMD-21024 was low-pass filtered to 15 Å and used as an initial model to auto-refine (*O* symmetry imposed) these particles to yield a reconstruction at 4.2 Å resolution according to the FSC cutoff at 0.143. After grouping by image shift, CTF refinement (per-particle defocus, per-micrograph astigmatism and beam tilt) and 3D refinement (*O* symmetry imposed) the resolution improved to 2.9 Å at a FSC cutoff of 0.143. A round of 3D classification (*O* symmetry imposed, six classes, Tau = 4, 30 iterations) and subsequent class selection by visual inspection yielded a smaller particle set (683 154 particles) which, after 3D refinement (*O* symmetry imposed), CTF refinement, Bayesian polishing and further CTF refinement, yielded the final reconstruction at 2.3 Å resolution (FSC = 0.143).

#### 2.4.3. Apoferritin in 20%(*v*/*v*) glycerol at 300 keV

We used *RELION* 3.1 for data analysis of apoferritin in the presence of 20% glycerol (Supplementary Fig. S3). We aligned dose-weighted movie frames using *MotionCor*2 from within *RELION* 3.1, and estimated the initial CTF parameters using *CTFFIND*4 from within *RELION* 3.1. We manually picked 277 particles from five randomly selected micrographs, extracted the particles binned by 4 (4.12 Å per pixel, 48 pixel box size) and ran 2D classification requesting a single class. This class was used as a template for template picking, resulting in ∼1.8 million picks that were extracted binned by 4 (4.12 Å per pixel, 48 pixel box size) and subjected to 2D classification (50 classes, Tau = 2, 25 iterations). The selection of 19 high-quality 2D classes in which secondary structure was visible yielded a particle stack of 778 762 particles from 1859 micrographs. An initial model generated in *cryoSPARC* version 2.14.2 (Punjani *et al.*, 2017 ▸) during previous exploratory work was used as a reference model for 3D auto-refinement (*O* symmetry imposed) of the particle stack. As 3D classification failed to generate distinguishable classes, the whole particle stack was re-centered and re-extracted at full resolution (1.03 Å per pixel, 192 pixel box size). Refinement (*O* symmetry imposed) of the unbinned stack resulted in a reconstruction at a nominal resolution of 2.9 Å at FSC = 0.143. The particles were grouped by image shift and iteratively CTF-refined with beam-tilt correction and per-particle defocus refinement. Subsequent auto-refinement (*O* symmetry imposed) improved the nominal resolution to 2.1 Å at FSC = 0.143.

We searched for densities that might correspond to ordered glycerol molecules, but were unable to unambiguously identify any. To carry out this analysis, we refined an existing atomic model of apoferritin into the density using the *Phenix* 1.19.2-4158 software suite (Liebschner *et al.*, 2019 ▸), beginning by applying anisotropic sharpening with default settings to produce a map from the final reconstructed half maps. We next docked model 1 from PDB entry 6v21 (Wu *et al.*, 2020 ▸), excluding nonprotein atoms, into the density and carried out real-space refinement with default settings. We manually inspected each residue from the resulting model in *Coot* (Emsley *et al.*, 2010 ▸). We placed Na^+^ ions at locations where positive ions had previously been modeled (Wu *et al.*, 2020 ▸) and placed water molecules using *phenix.douse* (Liebschner *et al.*, 2019 ▸) with default settings. We then carried out another round of real-space refinement. We searched for unmodeled blobs using the *Coot* validation node and a setting of 2 r.m.s.d. for ‘Find blobs above’, but none of the blobs found in this way could be confidently modeled as glycerol molecules.

#### 2.4.4. Aldolase in 20%(*v*/*v*) glycerol at 200 keV

We used *RELION* 3.1 and *Appion* (Lander *et al.*, 2009 ▸) for data analysis of aldolase in the presence of 20% glycerol (Supplementary Fig. S4). We aligned dose-weighted movie frames using *MotionCor*2 from within *RELION* 3.1 and then imported them to *cryoSPARC* for 2D analyses. Subsequent 3D classification and refinement was performed in *RELION* 3.1. We initially selected particles using *DoG Picker* 2 (Voss *et al.*, 2009 ▸) on the initial 76 micrographs after they had been aligned and dose-weighted in real time using the *Appion* launcher. These particles were extracted and used as input to the *Appion* iterative multivariate statistical analysis/multi-reference projection routine to create 2D class averages for template-based particle picking using *FindEM* (Roseman, 2004 ▸) on all 741 micrographs. The resulting 323 571 particle coordinates were imported to *RELION* 3.1 and extracted binned by 4 (3.64 Å per pixel) in a final box of 64 pixels. We performed four successive runs of 2D classification (50 classes, Tau = 1, *E*-step resolution limited to 8 Å, 30 iterations) at each iteration, discarding only class averages that clearly did not contain particles. We re-centered and extracted the remaining 322 648 particles binned by 2 (1.82 Å per pixel) in a 128-pixel box and performed one more round of 2D classification, where we only kept classes where secondary-structure elements were visible and radial streaks from noise were minimal. We re-centered and re-extracted (no binning, 256 pixel box) this 142 300-particle set, subjected it to 3D auto-refinement (*D*2 symmetry imposed) starting from EMD entry EMD-8743 low-pass filtered to 15 Å, and obtained a reconstruction of aldolase with a reported resolution of 4.8 according to FSC = 0.143. Grouping by image shift and subsequent runs of CTF refinement, firstly with refinement of beam tilt only and subsequently with refinement of defocus and astigmatism, followed by auto-refinement, yielded a reconstruction at 4.2 Å resolution (FSC = 0.143). We performed 3D classification without alignment (four classes, Tau = 8, 25 iterations) and selected particles from the highest resolution class. 3D auto-refinement using these 28 588 particles followed by CTF refinement (defocus, astigmatism and beam tilt) improved the resolution after auto-refinement (*D*2 symmetry imposed) to 4.0 Å. Bayesian polishing followed by CTF refinement (defocus, astigmatism, beam tilt and trefoil) yielded a final reconstruction after auto-refinement (*D*2 symmetry imposed) at 3.3 Å resolution (FSC = 0.143).

#### 2.4.5. Aldolase in 20%(*v*/*v*) glycerol at 300 keV

We used *RELION* 3.1 and *cryoSPARC* version 3.1.0 (Punjani *et al.*, 2017 ▸) for data analysis of aldolase in the presence of 20% glycerol (Supplementary Fig. S4). We aligned dose-weighted movie frames using *MotionCor*2 from within *RELION* 3.1 and then imported them to *cryoSPARC* for 2D classification; subsequent 3D classification and refinement were performed in *RELION* 3.1. We used the pyem library (Asarnow *et al.*, 2019 ▸) to convert *cryoSPARC* particle data output files to *RELION* 3.1 STAR files. We imported the aligned and dose-weighted micrographs in *cryoSPARC*, ran patch CTF estimation and selected micrographs containing CTF estimates reporting signal at 5 Å resolution or better (1870 of a total of 2591 micrographs). We then performed template picking using low-pass filtered projections of EMD entry EMD-8743 as templates, yielding 1 255 598 particles that were extracted and input to *cryoSPARC* 2D classification. We performed two rounds of 2D classification. After the first run of 2D classification we selected all particles from classes that remotely resembled aldolase, even if the classes did not contain high-resolution features. The particles comprising these classes were used for a subsequent 2D classification run, after which only particles that were contained in classes bearing discernible secondary-structure elements were selected. The coordinates of these 143 234 particles were re-centered and re-extracted without binning with a 256-pixel box size using *RELION* 3.1. The initial refinement from these particles yielded a reconstruction at 6.5 Å resolution (FSC = 0.143). We then removed duplicates and re-centered and re-extracted the particles, after which 3D auto-refinement yielded a reconstruction at 6.0 Å resolution. We then performed 3D classification without alignment (six classes with Tau = 6), from which we isolated a single class where secondary-structure elements were discernible and radial streaking was minimal. This class contained 21 155 particles. Refinement using this particle set yielded a 5.6 Å resolution reconstruction. Grouping by image shift followed by beam-tilt refinement and 3D auto-refinement improved the resolution to 5.3 Å, and a final refinement round using ‘Mask individual particles with zeros = No’ yielded a reconstruction at 4.5 Å resolution (FSC = 0.143).

### 2.5. Accrued beam-induced motion calculations

To calculate the accrued beam-induced motion, we used the global shift data collected in the STAR files produced by *RELION* 3.1 after running *MotionCor*2 from within the *RELION* 3.1 interface. Only micrographs containing particles from the final refinement set were used for this analysis [1858 out of 2261 for the apoferritin in 20%(*v*/*v*) glycerol data set collected at 200 keV, 1434 out of 1707 for the apoferritin in 1.7%(*v*/*v*) glycerol data set collected at 200 keV, and 735 out of 741 for the aldolase in 20%(*v*/*v*) glycerol data set collected at 200 keV]. To obtain the accrued motion per frame, we calculated the distance between the location in the current frame and in the previous frame and summed it to the total previous traveled distance. The dose at each data point is calculated as the dose per frame multiplied by the frame number.

### 2.6. Cryo-electron tomography data acquisition

Tilt series for apoferritin and aldolase samples were collected using a Thermo Fisher Titan Krios TEM at 300 keV equipped with a Gatan K2 Summit direct electron detector. Tomography data were acquired on a small region of the grid which was later avoided for single-particle data collection. Data acquisition was performed using *SerialEM* (Mastronarde, 2003 ▸). Tilt series were acquired using a sequential tilting scheme, starting at 0° and increasing to +60° in 2° increments, then returning to 0° and increasing to −60° in 2° increments. Each tilt series was collected with a nominal defocus of −6 to −8 µm for apoferritin and −10 µm for aldolase samples. Each tilt was acquired as movies in counting mode using a per-frame dose of 0.9 e^−^ Å^−2^ and a pixel size of 3.73 Å (13 404×), targeting holes randomly positioned within the chosen squares. For aldolase, we acquired tilt series on two sets of six holes each, arranged on two transects on neighboring grid squares where particles were visible under the imaging conditions used for single-particle data acquisition. For apoferritin, we acquired seven tilt series on a sample without added glycerol and 14 tilt series on a sample with glycerol in the buffer, on squares that were not used for single-particle data collection but were of similar quality. For both apoferritin and aldolase samples imaged at 300 keV, ice-thickness data were gathered on the same grids from which single-particle data were subsequently acquired. The squares selected for ice-thickness measurements were chosen to match the transparent ice area of those imaged for single-particle data.

### 2.7. Cryo-electron tomography data analysis

Image stacks from apoferritin and aldolase were binned by a factor of four prior to tomogram alignment using the *newstack* application from *IMOD* (Kremer *et al.*, 1996 ▸). Tomogram reconstruction was performed in the *eTomo* (Mastronarde, 1997 ▸) module of *IMOD*, following the pipeline from coarse alignment to fine and final alignments. Ice-thickness measurements from reconstructed tomograms were performed as described previously (Noble *et al.*, 2018 ▸). In brief, the binned tomograms were oriented in ‘3dmod slicer view’ in *IMOD* for optimal viewing of the *X–Z* axis, with the top and bottom particle layers (*i.e.* the air–water interface) roughly parallel to the field of view. These particle layers on the air–water interface were used as indicators of the ice boundary. Occasionally, ice contamination deposited on the ice surface further confirmed the location of the ice boundary. The 3*dmod* *IMOD* module was used to measure ice thickness at the hole edges and in the center, as delineated by the distance between the top and bottom particle layers.

The particle space-filling models embedded in ice shown in Fig. 2 and Supplementary Fig. S9 were created using *Dynamo* (Castaño-Díez *et al.*, 2012 ▸). The final density obtained by single-particle analysis of the same sample was used as a template for template matching in the aligned tomograms using *Dynamo*. In order to prepare the density for use as a template, we inverted the contrast using the *EMAN*2 (Tang *et al.*, 2007 ▸) e2proc3d.py script and down-sampled to the same pixel size as the tomograms using the *RELION* 3.1 (Zivanov *et al.*, 2018 ▸) auxiliary application *relion_image_handler*. A surface rendering of the same density, generated with *Dynamo*, was then placed at each cross-correlation peak in the tomogram volume only in locations where the cross-correlation values were above 0.33. To verify that the depicted particles were not located at correlation maxima derived from noise, we plotted the raw tomogram data of 100 randomly selected particles and visually confirmed that they resembled apoferritin.

### 2.8. Direct plunging in liquid nitrogen of an apoferritin sample in the presence of 20% glycerol

The grids were plasma-cleaned and protein samples were prepared as described above. Blotting was performed from the side using Whatman No. 1 filter paper, as normally performed for negative staining. Plunging was performed by hand, simply submerging the grids completely in liquid nitrogen. These frozen-hydrated samples were imaged at liquid-nitrogen temperature on a Tietz 4k × 4k CCD camera with a defocus of −2.5 µm using the *Leginon* automated electron-microscopy package with an FEI F20 electron microscope operating at 200 keV at a nominal magnification of 62 000× (a pixel size of 1.774 Å).

### 2.9. CryoEM map rendering and local resolution coloring

Electron-potential maps were rendered using *UCSF Chimera* (Pettersen *et al.*, 2004 ▸). Local resolution calculations were performed in *RELION* 3.1 and the outputs were used for local resolution coloring in *UCSF Chimera*.

## 3. Results

### 3.1. High-resolution structure determination of apoferritin in the presence of 20% glycerol imaged at 200 keV

**Figure 1:**
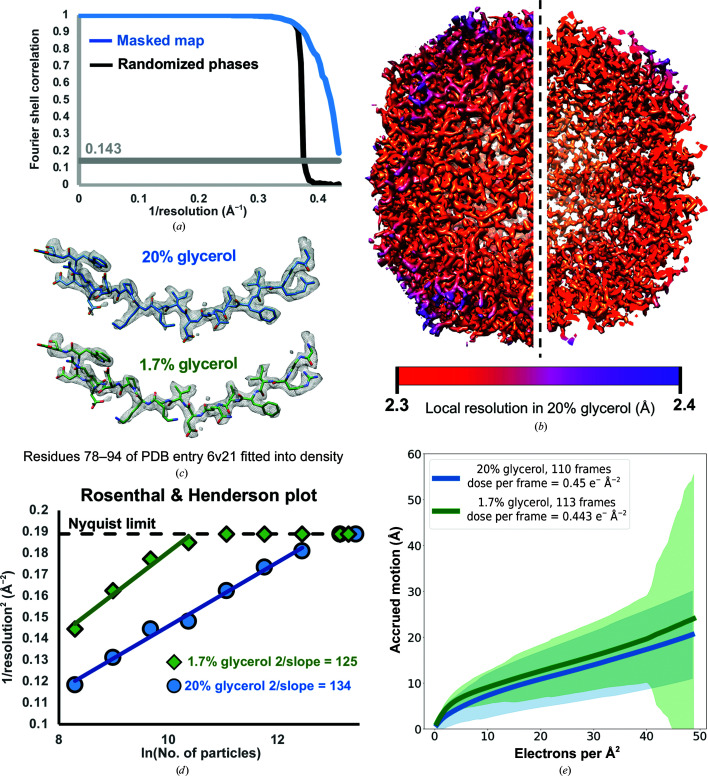
Apoferritin reconstructions acquired at 200 keV in the presence of 1.7% and 20% glycerol. (*a*) Fourier shell correlation (FSC) between masked half maps (blue line) and between phase-randomized half maps (black line) from the apoferritin reconstruction in the presence of 20% glycerol. The gray line shows the gold-standard (0.143) correlation value. (*b*) Reconstruction of apoferritin in the presence of 20% glycerol, colored by local resolution. The right half of the image is a cutaway showing the interior. (*c*) Apoferritin model (PDB entry 6v21) fitted into the EM density from reconstructions in 20% (blue) and 1.7% (green) glycerol. (*d*) *B*-factor plot (Rosenthal & Henderson, 2003 ▸) showing the improvement of resolution as a function of the number of particles used for reconstruction in the presence and absence of a high glycerol concentration. (*e*) Accrued beam-induced motion (per-frame summation) as a function of the number of electrons per Å^2^ received by the sample. Solid lines: average over all collected movies at a given dose. Colored shades: standard deviation over all movies at a given dose.

In order to determine the effect that glycerol has on the resolution of structures that can be obtained using single-particle analysis, we acquired data on mouse heavy-chain apoferritin in the presence of 1.7%(*v*/*v*) and 20%(*v*/*v*) glycerol using a 200 keV Talos Arctica. The protein apoferritin assembles into an ∼500 kDa octahedron and is widely used for cryoEM studies due to its size and stability; it has been resolved to better than 2 Å resolution in previous studies (Yip *et al.*, 2020 ▸). Notably, the inclusion of 20% glycerol did not impede our ability to determine high-resolution structures, as both data sets (with or without added glycerol) gave rise to reconstructions of apoferritin with reported resolutions at the Nyquist frequency (2.3 Å; Fig. 1 ▸ *a* and Supplementary Fig. S7*d* ). Moreover, the densities of both reconstructions show a tight local resolution distribution near 2.3 Å, not surpassing 2.4 Å (Fig. 1 ▸ *b* and Supplementary Fig. S7*f*), and the density quality and detail of local features are indistinguishable between the two reconstructions (Fig. 1 ▸ *c*). However, the higher glycerol concentration indeed appeared to decrease the overall quality of the data, as more extensive processing was involved in attaining the final structure (Supplementary Figs. S1 and S2), a greater number of particles were required to achieve high resolution (consistent with the higher *B* factor and significantly lower *y*-axis intercept shown in the Rosenthal and Henderson plot; Fig. 1 ▸ *d*) and the estimated accuracy of angles was lower (0.24° in 1.7% glycerol versus 0.37° in 20% glycerol). These data indicate that while glycerol does not necessarily prevent the ability to resolve a high-resolution reconstruction of a large, symmetric and stable biological complex, it nonetheless negatively impacts the data quality.

### 3.2. High concentrations of glycerol prevent the formation of optimally thin ice, lowering contrast

**Figure 2:**
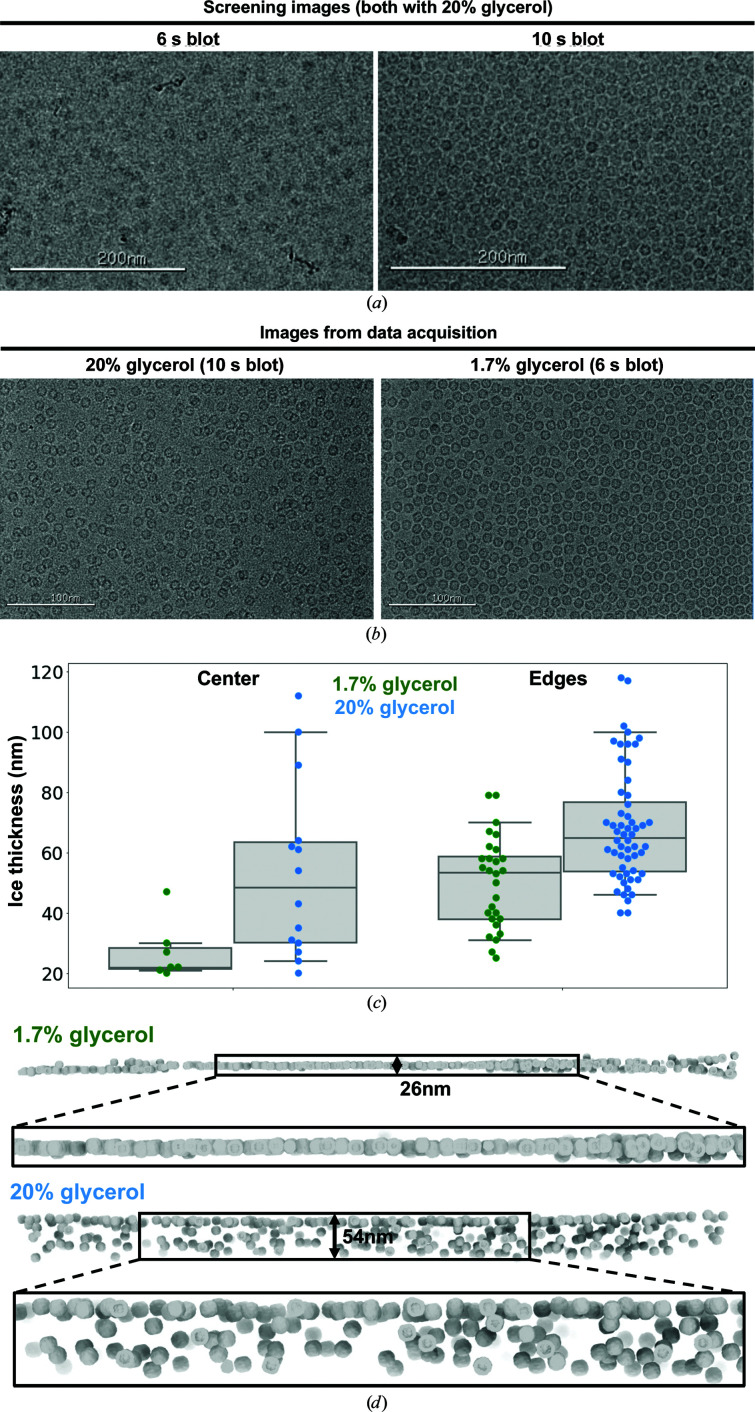
Cryo-electron tomography of apoferritin samples acquired in the presence of 1.7% and 20% glycerol. (*a*) Electron micrographs from sample screening manually blotted for different times. (*b*) Exemplar micrographs of samples with 1.7% and 20% glycerol. (*c*) Ice-thickness measurements obtained by electron tomography. Data points are shown as colored dots (horizontally stacked only to aid clarity) overlaid on a gray box that spans the two central quartiles and is crossed by a vertical line at the median. The whiskers span the range of data between the 5th and 95th percentiles. (*d*) 3D renderings of ice cross-sections from representative micrographs in samples with and without added glycerol, generated by matching an apoferritin density template on the aligned tomograms and placing oriented density surface renderings as markers at high cross-correlation locations.

These two data sets offered an opportunity to perform a quantitative characterization of the impact that glycerol has on cryoEM data collection and to identify the sources of data-quality degradation. Notably, there was no indication of increased radiation damage in the form of bubbling present in any of the micrographs containing 20% glycerol, nor did the accrued motion significantly differ between the samples with or without added glycerol (Fig. 1 ▸ *e*). However, the images acquired in the presence of 20% glycerol showed a qualitatively lower contrast than the samples with no added glycerol. We suspected that the likely source of this lowered contrast was the apoferritin particles being embedded in a thicker layer of ice when glycerol is present. The increased viscosity of the 20% glycerol sample was evident during the grid-preparation process, as the blotting time required to obtain ice that was thin enough for data acquisition was increased nearly twofold for the 20% glycerol sample (Figs. 2 ▸ *a* and 2 ▸ *b*). A longer blotting time is generally associated with thinner ice, as the filter paper absorbs an increasing volume of sample solution. Despite this longer blotting time, we were unable to attain sufficiently thin ice to produce a monolayer of apoferritin across the holes in the presence of 20% glycerol. This is showed by the presence of overlapping apoferritin particles in the micrographs and 2D averages (Fig. 2 ▸ *b* and Supplementary Fig. S6).

We further characterized the difference in ice thickness between the samples of apoferritin with and without added glycerol using cryo-electron tomography. Newly prepared apoferritin samples containing 1.7% and 20% glycerol were loaded into a Titan Krios operating at 300 keV and tomograms of the grid holes were acquired (seven tomograms of the 1.7% glycerol sample and 14 tomograms of the 20% glycerol sample). Analysis of the tomograms revealed that the ice in the higher glycerol concentration samples was, on average, twice as thick. Notably, in the presence of 20% glycerol the ice thickness at the center of the hole is more variable in different holes, while at 1.7% glycerol the ice at the center of the holes was reproducibly thin (Fig. 2 ▸ *c*). Ideally, for single-particle cryoEM analyses the ice thickness should only be slightly thicker than the diameter of the targeted particle in order to minimize the contribution of buffer molecules to the detected image. For this reason, images are routinely acquired at the center of the grid holes, where the ice is generally thinnest and particles are often arranged as a single monolayer. In the presence of 1.7% glycerol, particles are mostly located in a single layer between the two surfaces of the air–buffer interface. In contrast, we identified many fewer regions with comparably thin ice on the grid containing the 20% glycerol sample.

Intriguingly, the tomograms of the 20% glycerol grid confirmed that apoferritin particles were located throughout the vitrified ice within the holes, as opposed to almost exclusively being adsorbed to the air–water interfaces, as observed previously in the absence of glycerol (Noble *et al.*, 2018 ▸). The percentage of particles located away from the air–water interface in our sample appears to be higher than described for samples without glycerol with a comparable ice thickness (see the description of sample 36 and the movie in Noble *et al.*, 2018 ▸). This may be due to the increased viscosity of the buffer limiting the mobility of the particles during the blotting process or perhaps diminishing the hydrophobic interactions that promote adhesion of particles to the air–water interface. While this may benefit the overall particle stability, this also results in the appearance of multiple overlapping layers of particles when viewed along the optical axis (Fig. 2 ▸ *d* and Supplementary Figs. S6 and S9). Micrographs for single-particle analysis were also acquired on the 20% glycerol sample that was used for tomographic data collection, and processing these data yielded a reconstruction with a reported resolution at the Nyquist frequency (2.1 Å resolution; Supplementary Fig. S8). An atomic model based on this density was practically identical to PDB entry 6v21 (Wu *et al.*, 2020 ▸; all-atom r.m.s.d. of 0.22 Å). Despite containing a high level of structural detail, which enabled the confident modeling of water molecules, no glycerol molecules could be placed confidently as all of the unmodeled density lacked sufficient detail.

These data provide an explanation for the higher *B* factors and lower *y* intercept associated with the 20% glycerol sample. The increased ice thickness increases the likelihood of electron scattering due to interactions with the buffer molecules, detracting from the particle contrast in the images. The distribution of particles at different heights within the ice also results in the appearance of overlapping particles in the images, which can complicate the accurate assignment of alignment parameters to each particle. Furthermore, the presence of particles at multiple heights in thick ice results in a distribution of defocus values corresponding to the particles, which decreases the accuracy of CTF estimation. Given the close proximity of particles to one another, it is also unlikely that per-particle CTF estimators will be able to assign defocus values to individual particles with high accuracy.

### 3.3. Reconstruction of rabbit muscle aldolase at ∼3.3 Å resolution in the presence of glycerol

**Figure 3:**
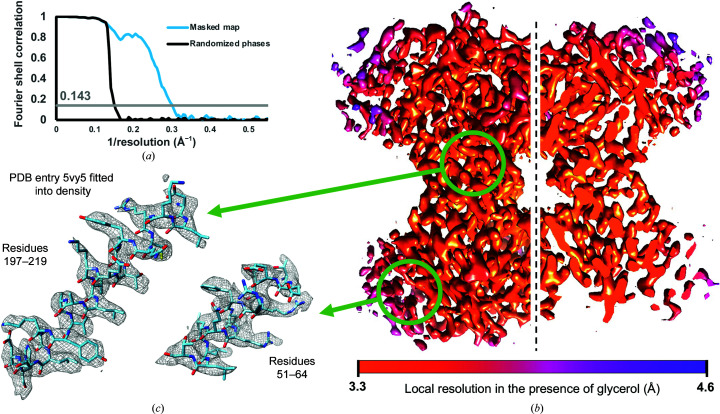
Reconstruction of aldolase at 300 keV in the presence of 20% glycerol. (*a*) Fourier shell correlation between masked half maps (blue line) and between phase-randomized half maps (black line) from the aldolase density reconstruction in the presence of 20% glycerol. The gray line shows the 0.143 gold-standard correlation value. (*b*) Density reconstruction of aldolase from data acquired in the presence of 20% glycerol, colored by local resolution. (*c*) Enlarged segments of aldolase density reconstruction from two areas with different local resolution. PDB entry 5vy5 was aligned with the density and the corresponding atoms are shown inside the mesh.

Our apoferritin tests demonstrated that although glycerol increased the ice thickness, thereby decreasing the overall quality of a single-particle data set, we were nonetheless able to determine a high-resolution structure of a stable, symmetric, 500 kDa protein complex. We next set out to test whether the addition of glycerol imposes resolution boundaries on smaller specimens, which are much more sensitive to ice thickness due to the lower overall electron scattering contribution of the biological specimen. To test the impact of 20% glycerol on a sample smaller than 200 kDa, we selected rabbit muscle aldolase, a protein that assembles into a 160 kDa homotetramer with *D*2 symmetry. Previously, we generated a reconstruction of this complex at ∼2.6 Å resolution using a 200 keV electron microscope, and to test the impact of glycerol we prepared the same sample with 20% glycerol using the same imaging parameters and microscope. Similarly to the apoferritin samples, a blotting time of 10 s was necessary to obtain sufficiently thin ice for data acquisition, which was ∼2 times longer than required for the sample in the absence of glycerol (Wu *et al.*, 2020 ▸). Despite a qualitatively noticeable decrease in contrast in our micrographs compared with those previously acquired without glycerol, we were able to obtain a resolution of ∼3.3 Å in the presence of 20% glycerol (Figs. 3 ▸ *a* and 3 ▸ *b* and Supplementary Fig. S10). Notably, the structural detail in our reconstruction was of sufficient quality to confidently assign side-chain identity in the core of the reconstruction (Fig. 3 ▸ *c*). Analysis of the micrographs and frame-alignment statistics showed that, as for apoferritin, the added glycerol did not result in radiation-induced bubbling or any significant increases in beam-induced motion (Supplementary Fig. S11).

We prepared additional aldolase grids with 20% glycerol to assess the ice thickness by tomography on the 300 keV Krios and confirmed that the ice was substantially thicker than that previously reported for aldolase prepared in the absence of glycerol (Noble *et al.*, 2018 ▸; Supplementary Fig. S12). Single-particle data collected from this grid yielded a reconstruction at 4.6 Å resolution (estimated accuracy of angles of 6.8°; Supplementary Fig. S13). It is unlikely that the difference in accelerating voltage was responsible for the difference in the attained resolution. Rather, the lower resolution of this reconstruction may stem from the lower dose used for imaging (50 e^−^ Å^−2^, which is close to the typical values used for single-particle imaging, instead of the 80 e^−^ Å^−2^ used in the 200 keV data set) and/or thicker ice than for the 200 keV aldolase data set, underscoring the challenge of consistently obtaining thin ice when glycerol is present at such high concentrations.

## 4. Concluding remarks

Our results indicate that despite presenting challenges for optimal ice formation, the introduction of glycerol molecules into the buffer does not abolish the ability to determine high-resolution structures of biological specimens using single-particle cryoEM. At the electron doses (50 and 80 e^−^ Å^−2^) and buffer compositions used in this study, we do not observe significant changes in beam-induced motion or radiation-induced bubbling for glycerol-containing samples compared with those with little or no glycerol. We do, however, confirm that the introduction of glycerol into the buffer lowers the overall quality of the data. We found that the lower contrast of glycerol-containing samples originates from the difficulty of generating thin ice compared with samples lacking glycerol. Such issues with ice thickness can also arise from additives other than glycerol, such as DMSO, and ice-thickness optimization is a necessary aspect of cryoEM sample preparation. Sample volumes, ambient humidity, blotting times and forces could all be optimized to attain an appropriate ice thickness. Self-wicking grids may be used in combination with small-volume dispensing equipment to obtain consistently thin ice in samples with high glycerol concentrations. In addition to finely controlling the dispensed volume, the application-to-freezing time can be tuned to control sample absorption by the grid nanowires (Dandey *et al.*, 2018 ▸). Of course, lower glycerol concentrations [for example 10%(*v*/*v*)] could potentially be used to attain thinner ice without substantially impacting macromolecular stability.

It is possible that the previously documented imaging complications attributed to glycerol originate from sample and/or imaging conditions that differ from current single-particle imaging practices. The single-particle community routinely images samples at higher voltages (200 and 300 keV) than those used in past literature (100 and 120 keV; Karuppasamy *et al.*, 2011 ▸; Frederik *et al.*, 1991 ▸). These lower voltages are known to cause higher rates of radiation damage (Peet *et al.*, 2019 ▸). Further, the glycerol-containing samples described in the previous literature consisted of much thicker ice films than those imaged for single-particle studies. For comparison, at the edge of our thickest samples the ice thickness was around ∼80 nm, while the 50%(*v*/*v*) glycerol sample described by Karuppasamy *et al.* (2011 ▸) was 150 nm thick. An additional consideration is that some of the previously characterized glycerol-containing samples consisted of thin films of collagen and lipid vesicles, and that the sections with higher collagen content began bubbling before other sections (Frederik *et al.*, 1991 ▸), suggesting that the combination of glycerol with large biomolecular aggregates may be detrimental for imaging.

It is important to note that an often-overlooked benefit of thicker ice is the loosened confinement of large or dimensionally anisotropic particles. It is common for large particles to retreat to the edges of holes, where they are better accommodated by thicker ice, while particles in the centers of the holes are either denatured/broken or forced to assume a preferred orientation that best accommodates the thinner ice. Thus, the limited thinning of ice offered by the addition of glycerol may aid in increasing the spatial and orientational distribution of particles in holes. Further, thicker ice may be preferable for fragile biological samples that denature at the air–water interface. Given that macromolecular targets requiring higher levels of glycerol for purification or functional assays are likely to fall into this category of fragile complexes, preservation in the thick ice promoted by 20% glycerol may be beneficial for structural stability and structure determination. Our tomograms show that the apoferritin particles are distributed throughout the vitrified buffer in holes, as opposed to being localized at the air–water interfaces, indicating that the addition of glycerol may protect a certain fraction of the sample from destructive hydrophobic inter­actions. Due to its small size, the same analysis was not possible for tomograms containing aldolase.

Lastly, we also note that direct plunging of an apoferritin sample containing 20% glycerol into liquid nitrogen seems to yield vitreous ice (Supplementary Fig. S14). As previously demonstrated by Taylor and Glaeser in the first studies of frozen-hydrated catalase crystals (Taylor & Glaeser, 1974 ▸), the cryoprotective qualities of glycerol enable vitrification of the sample without the need for plunging into a cryogen with high heat capacity such as ethane or propane. This observation could conceivably pave the way to simpler workflows in which the manipulation of fragile grids and ice contamination are minimized (Engstrom *et al.*, 2021 ▸).

We conclude that glycerol should not be discarded as a cryoEM sample-buffer additive, particularly for large, fragile complexes that are prone to disassembly or aggregation upon its removal. Further, the thicker ice and seemingly diminished interaction of particles with the air–water interface associated with the addition of glycerol may aid in promoting a wider range of orientations in ice. We expect these findings to encourage new structural studies of samples that were previously not considered to be amenable to cryoEM analysis.

## 5. Data accessibility

All reconstructions and associated micrographs have been deposited in the Electron Microscopy Data Bank (EMDB) and Electron Microscopy Public Image Archive (EMPIAR) and are accessible with the following IDs: apoferritin in 1.7% glycerol acquired at 200 keV, EMD-24795, EMPIAR-10857; apoferritin in 20% glycerol acquired at 200 keV, EMD-24796, EMPIAR-10849; apoferritin in 20% glycerol acquired at 300 keV, EMD-24797, EMPIAR-10861; aldolase in 20% glycerol acquired at 200 keV, EMD-24798, EMPIAR-10866; aldolase in 20% glycerol acquired at 300 keV, EMD-24799, EMPIAR-10867.

## 6. Related literature

The following reference is cited in the supporting information for this article: Tan *et al.* (2017 ▸).

## Supplementary Material

EMDB reference: apoferritin in 1.7% glycerol acquired at 200 keV, EMD-24795

EMDB reference: apoferritin in 20% glycerol acquired at 200 keV, EMD-24796

EMDB reference: apoferritin in 20% glycerol acquired at 300 keV, EMD-24797

EMDB reference: aldolase in 20% glycerol acquired at 200 keV, EMD-24798

EMDB reference: aldolase in 20% glycerol acquired at 300 keV, EMD-24799

Supplementary Figures and Tables. DOI: 10.1107/S2059798321012110/vo5007sup1.pdf
