# Supplementary material for: A case for glycerol as an acceptable additive for single-particle cryoEM samples
Source: Acta Crystallogr D Struct Biol. 2022 Jan 1;78(Pt 1):124–35. doi: 10.1107/S2059798321012110 (PMC8725161; doi:10.1107/S2059798321012110)
Supplement: Supplementary file 1 [file d-78-00124-sup1.pdf]

STRUCTURAL BIOLOGY

Volume 78 (2022)

Supporting information for article:

A case for glycerol as an acceptable additive for single-particle cryoEM samples

Benjamin Basanta, Marscha M. Hirschi, Danielle A. Grotjahn and Gabriel C. Lander

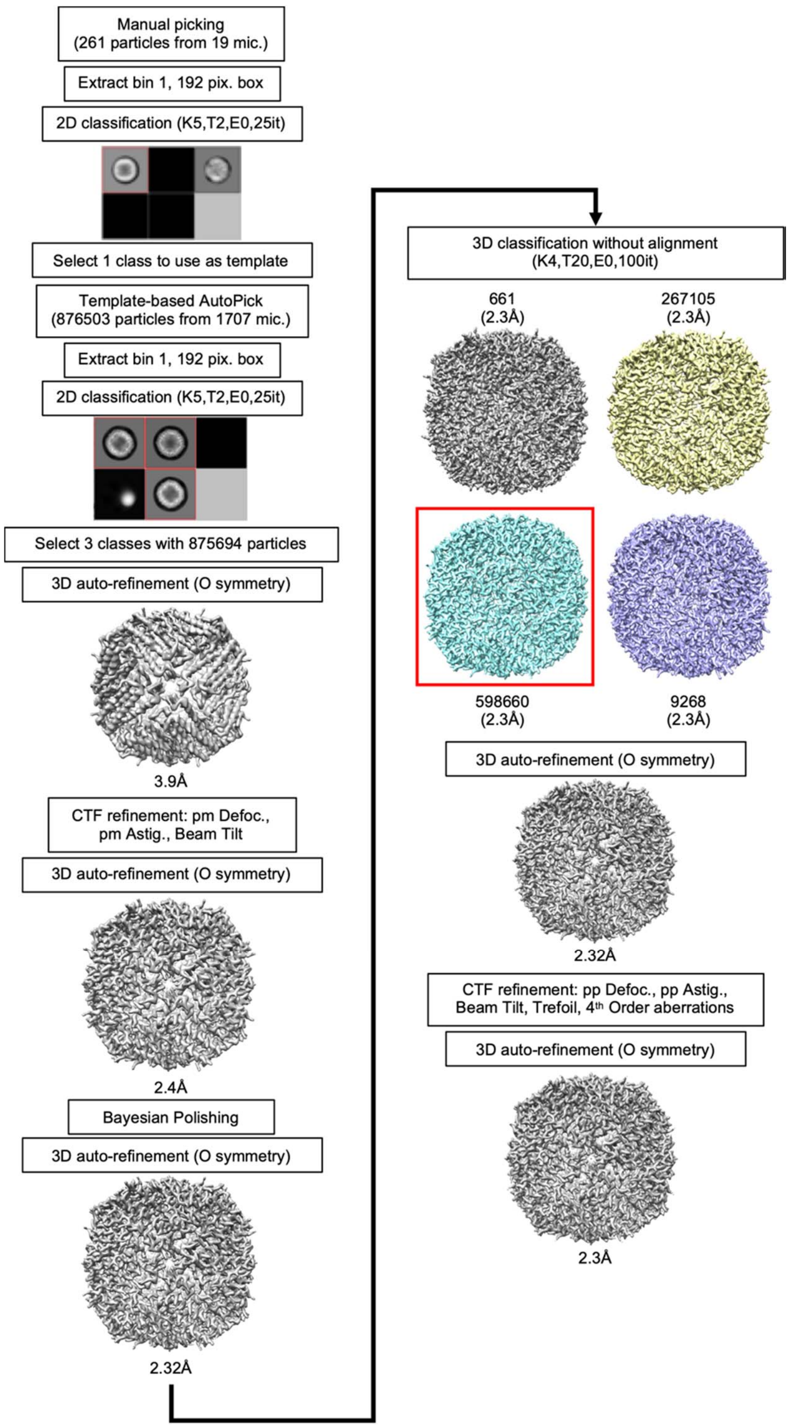

Figure S1 Data analysis workflow for apoferritin in 1.7% glycerol buffer imaged at 200KeV. In 2D and 3D classification steps, the selected classes are marked with a red box. Empty classes in 2D jobs are represented by dark squares. Relevant parameters for each RELION node are listed in each box.

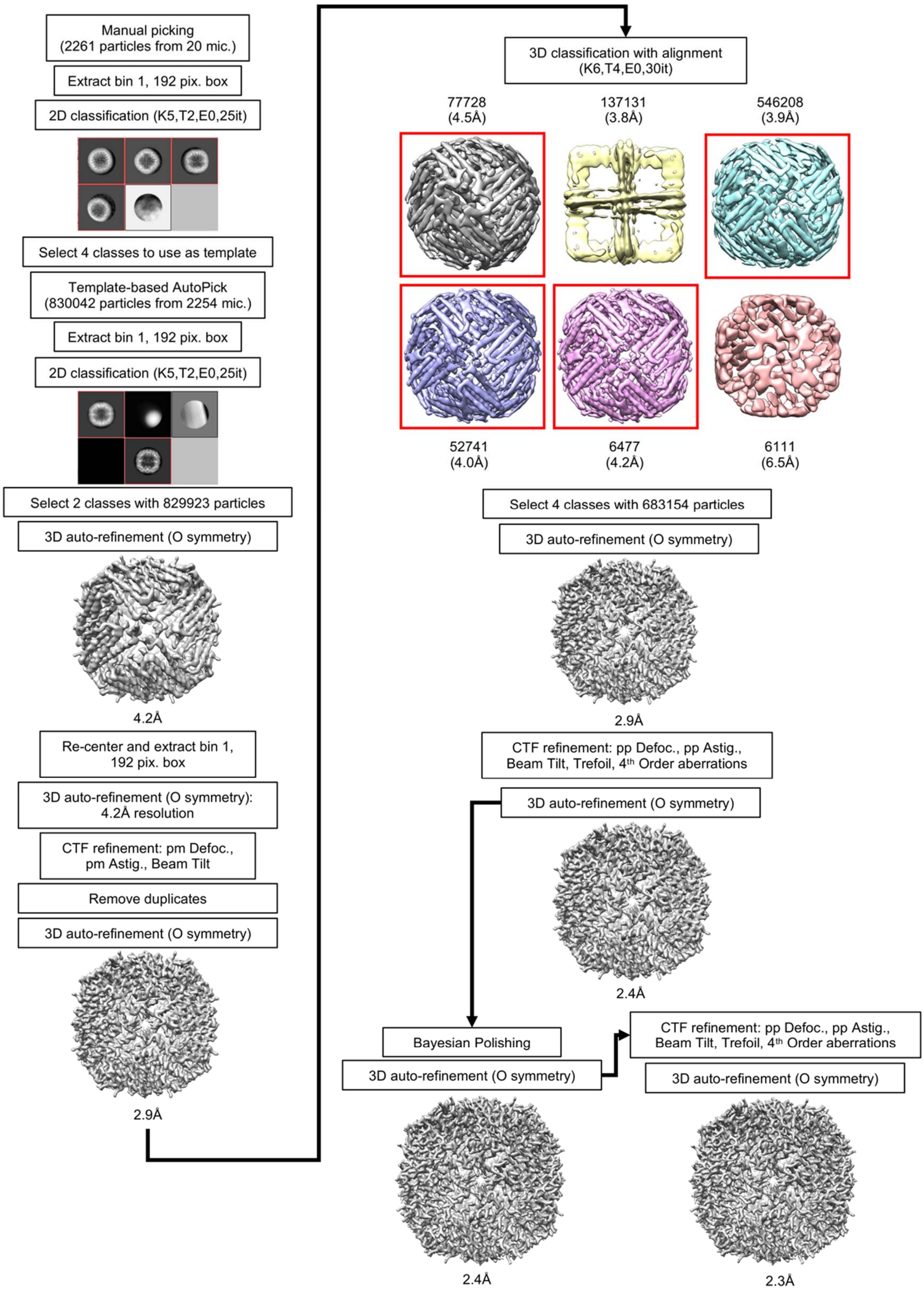

Figure S2 Data analysis workflow for apoferritin in 20% glycerol buffer imaged at 200KeV. In 2D and 3D classification steps, the selected classes are marked with a red box. Empty classes in 2D jobs are represented by dark squares. Relevant parameters for each RELION node are listed in each box.

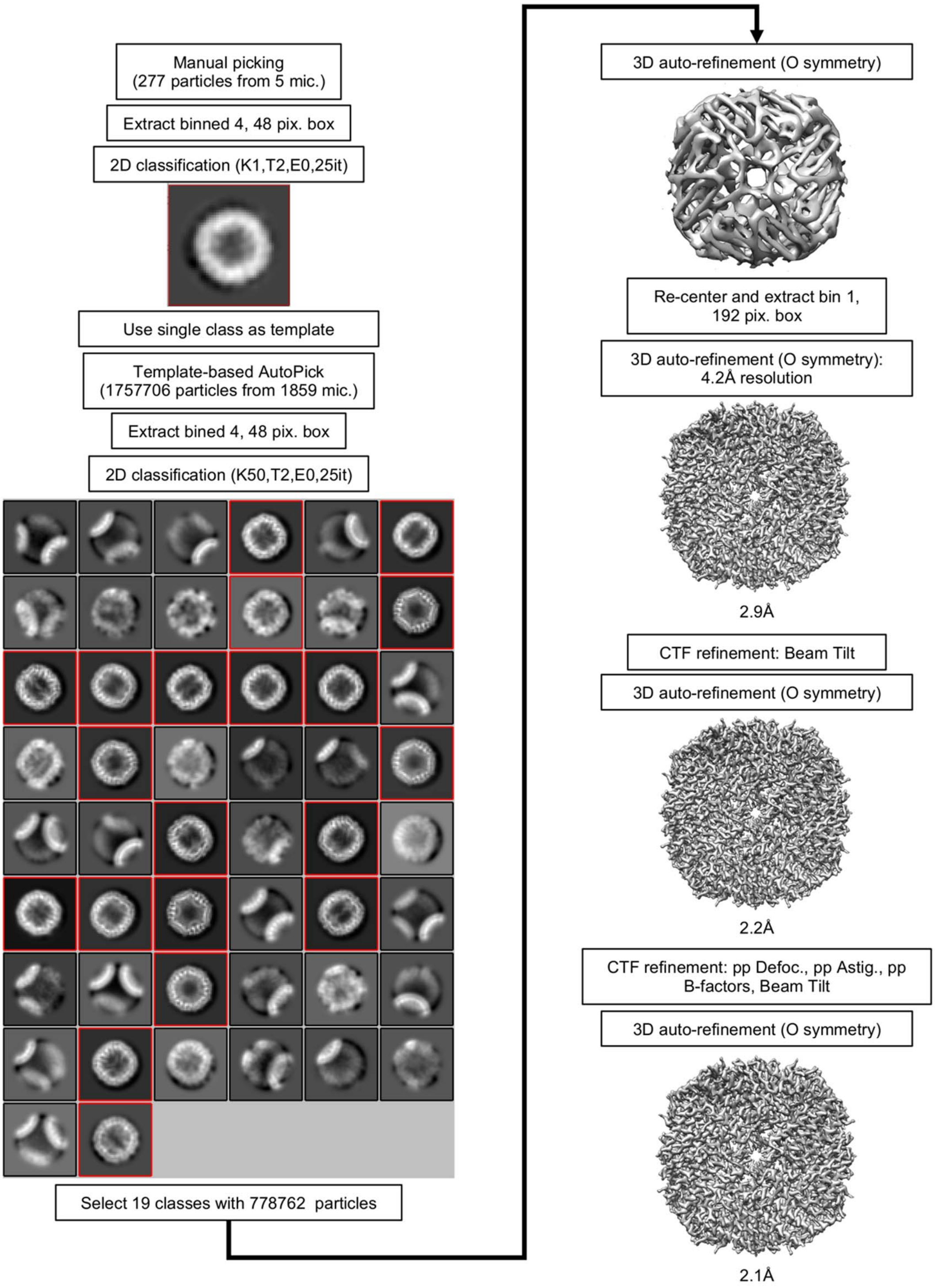

Figure S3 Data analysis workflow for apoferritin in 20% glycerol buffer imaged at 300KeV. In the 2D classification step, the selected classes are marked with a red box. Relevant parameters for each RELION node are listed in each box.

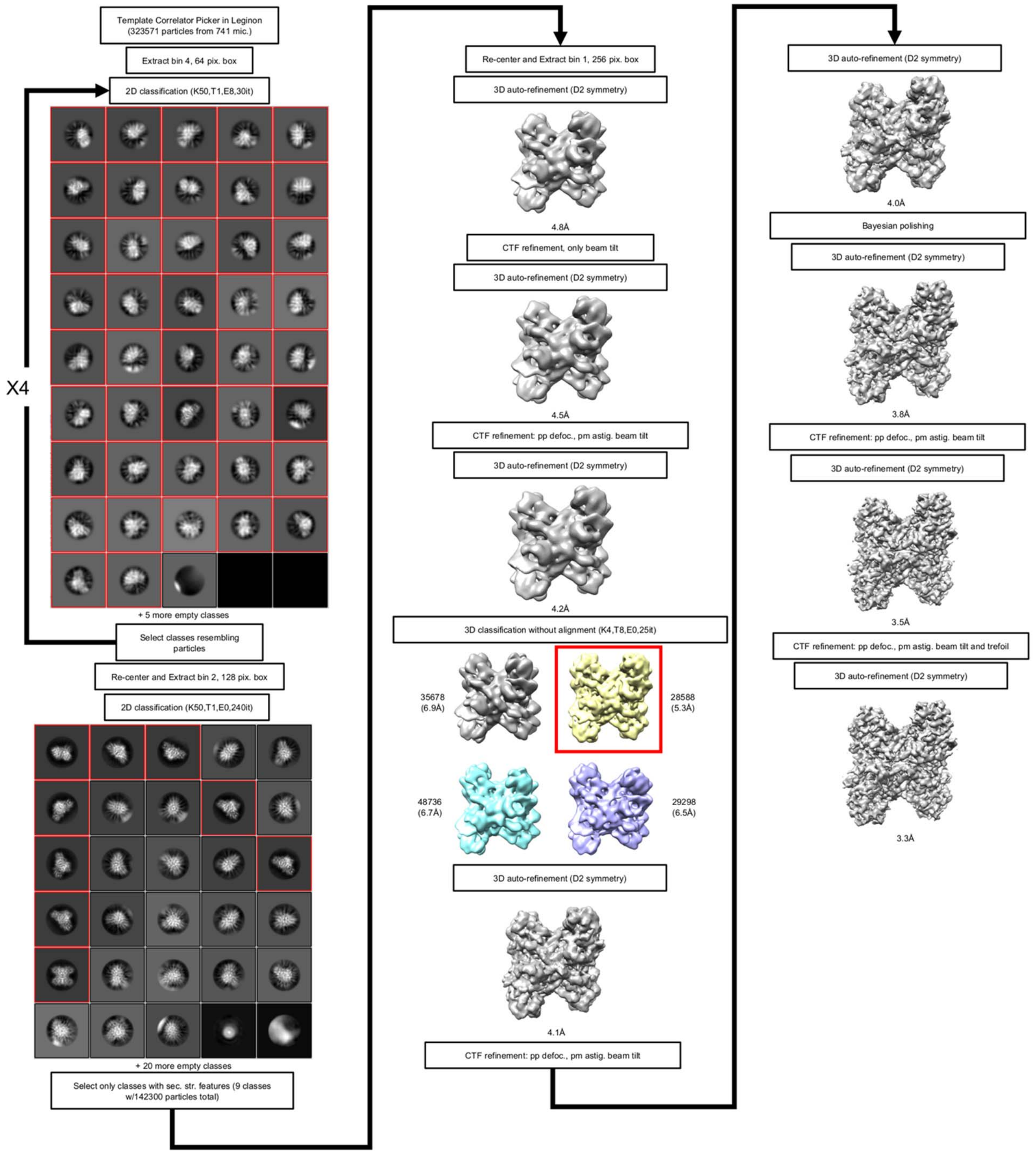

Figure S4 Data analysis workflow for aldolase in 20% glycerol buffer imaged at 200KeV. In 2D and 3D classification steps, the selected classes are marked with a red box. Empty classes in 2D jobs are represented by dark squares. Relevant parameters for each RELION node are listed in each box.

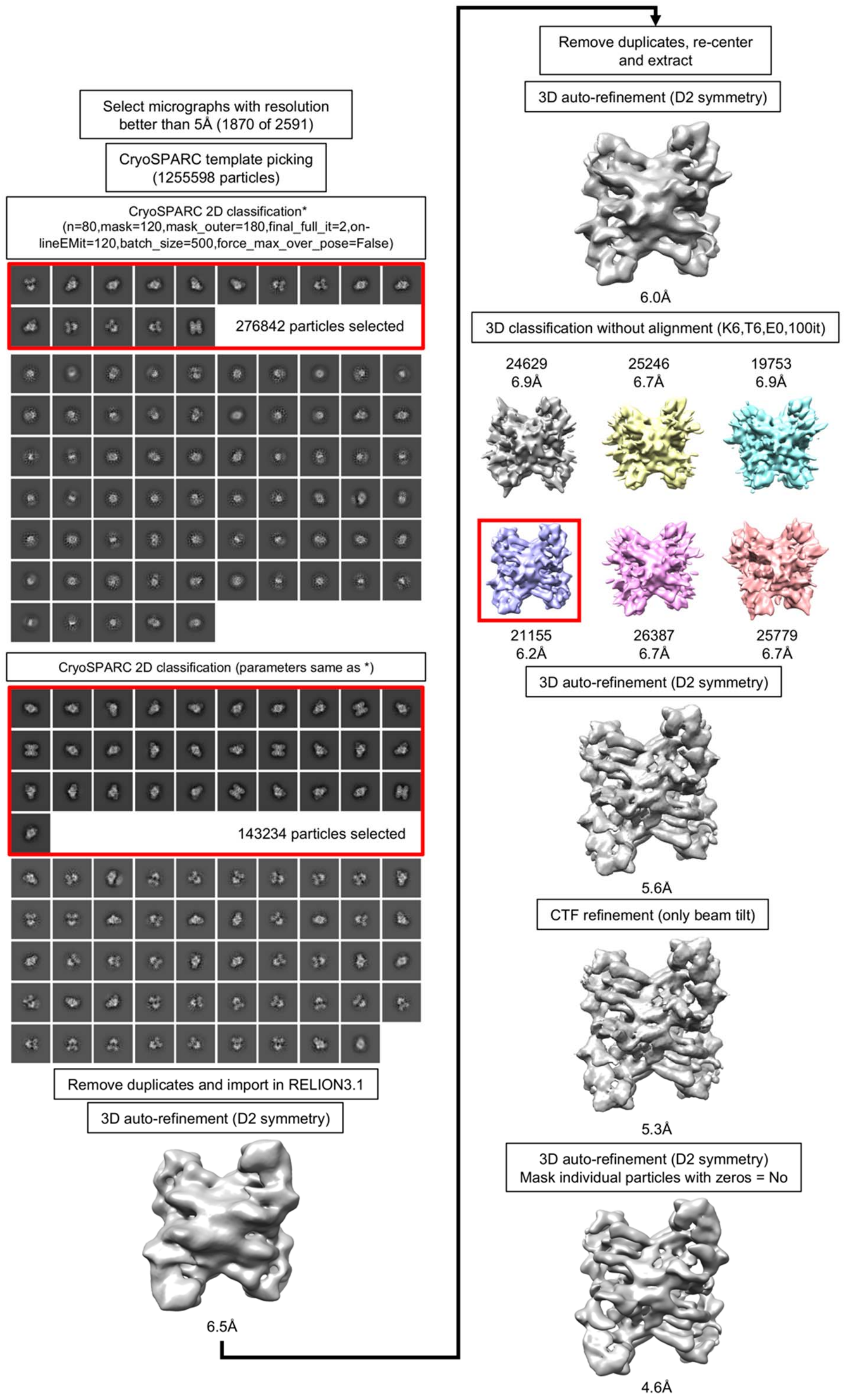

Figure S5 Data analysis workflow for aldolase in 20% glycerol buffer imaged at 300KeV. In 2D and 3D classification steps, the selected classes are marked with a red box. Empty classes in 2D jobs are represented by dark squares. Relevant parameters for each RELION node are listed in each box.

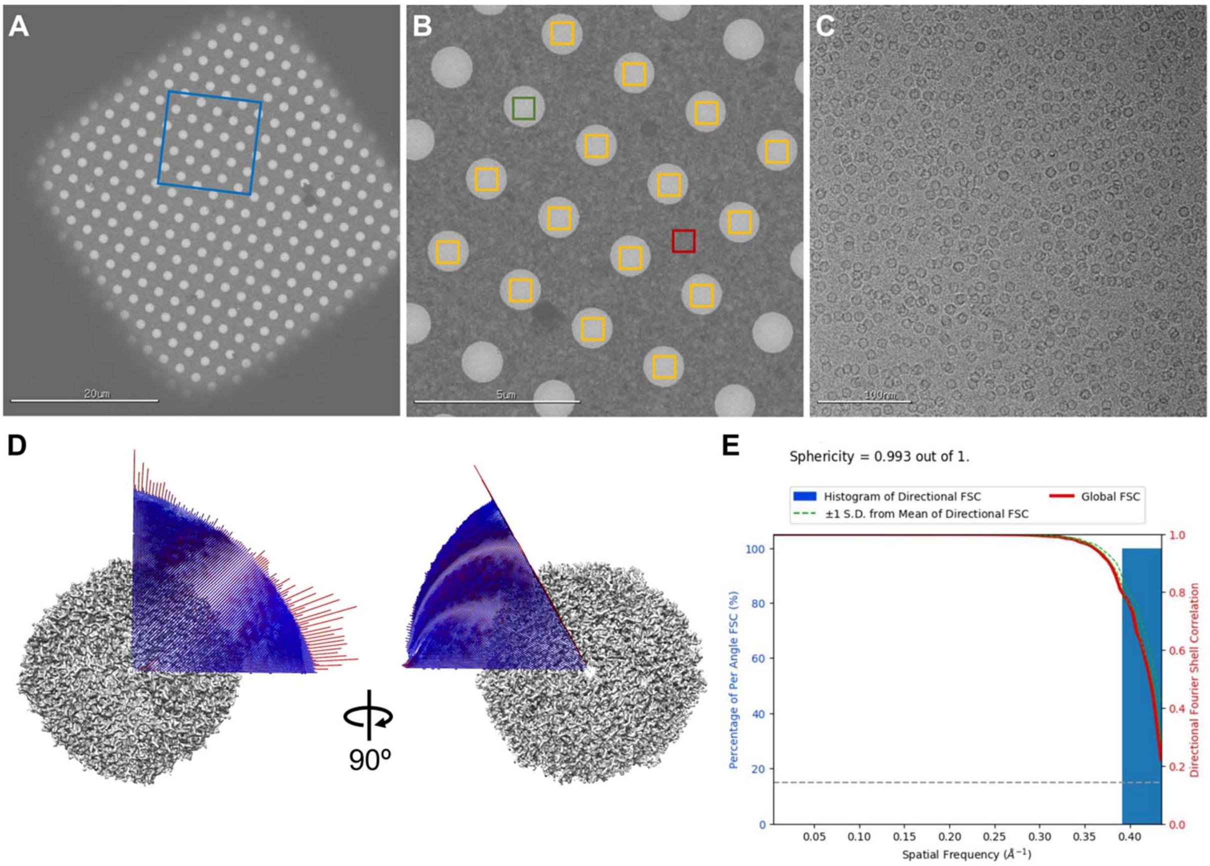

Figure S6 Exemplar micrographs and additional final reconstruction metrics for apoferritin imaged at 200KeV in 20% glycerol buffer. A. Exemplar grid square, a blue frame marks where micrograph B was acquired. B. An exemplar "hole" magnification micrograph, with a red square showing where real focus estimation was done, and yellow squares showing where high-magnification micrographs were acquired for single particle analysis. A green square shows where micrograph C was taken. C. An exemplar micrograph used for single particle picking and analysis. D. Angular distribution of views for the final reconstruction done assuming O symmetry. E. The global FSC curve (red) overlaid on a histogram of directional FSC curves (blue bars). Each bin in the directional FSC histogram indicates a spatial frequency bin at which the FSC curve crosses a correlation value of 0.143. The green dotted lines denote the standard deviation range of the correlations at each spatial frequency, as described in (Tan *et al.*, 2017).

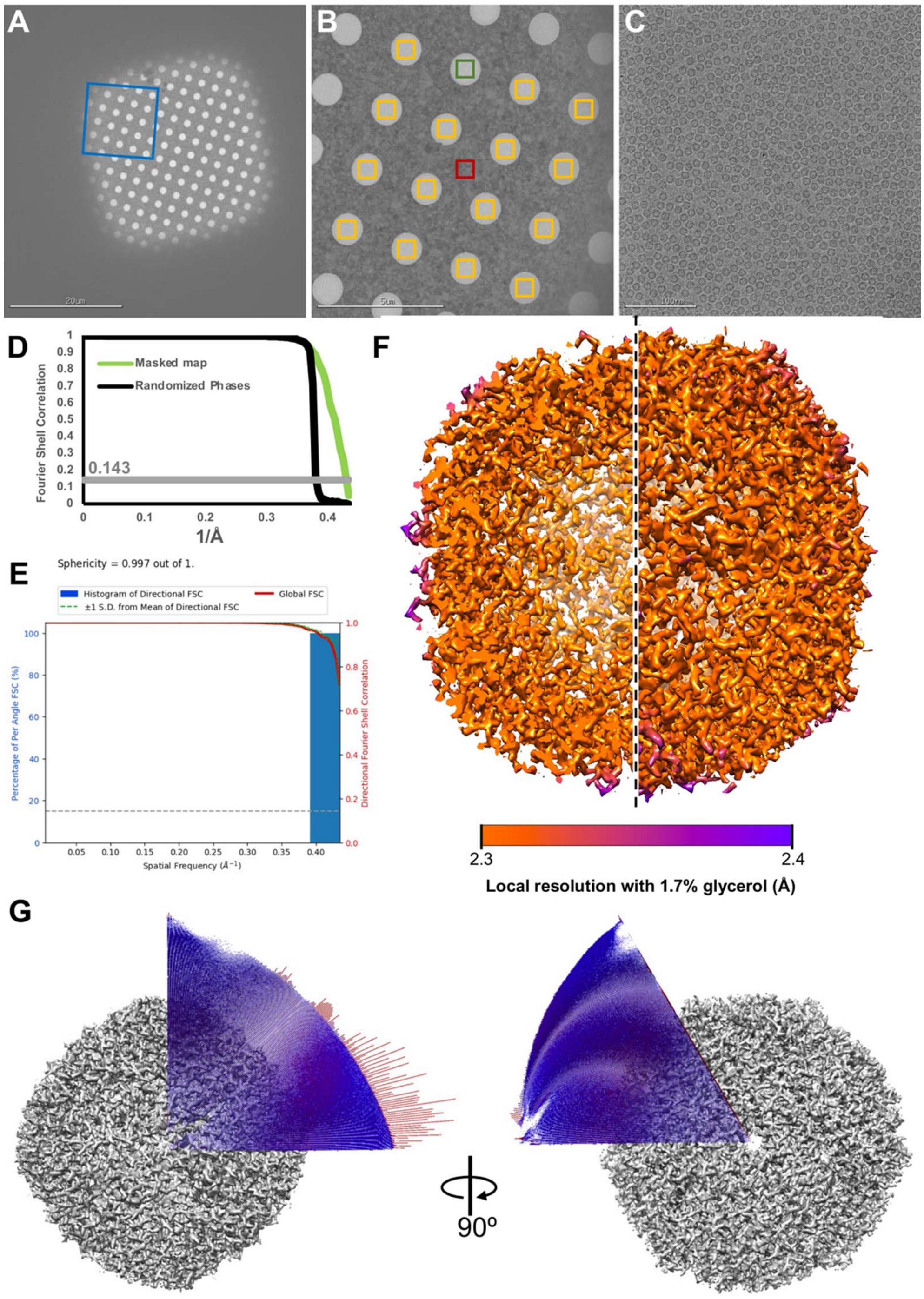

Figure S7 Exemplar micrographs and additional final reconstruction metrics for apoferritin imaged at 200KeV with 1.7% glycerol. A. Exemplar grid square, a blue frame marks where micrograph B was acquired. B. An exemplar "hole" magnification micrograph, with a red square showing where real focus estimation was done, and yellow squares showing where high-magnification micrographs were acquired for single particle analysis. A green square shows where micrograph C was taken. C. An exemplar micrograph used for single particle picking and analysis. D. Fourier shell correlation curve for the masked reconstruction and for randomized phases. E. Directional FSC curves (as described in

Supplementary Fig. 6E) showing how resolution varies by orientation (Tan *et al.*, 2017). F. Reconstruction colored by local resolution, calculated by RELION3.1 Local Resolution node (Zivanov *et al.*, 2018). G. Angular distribution of views for the final reconstruction done assuming O symmetry.

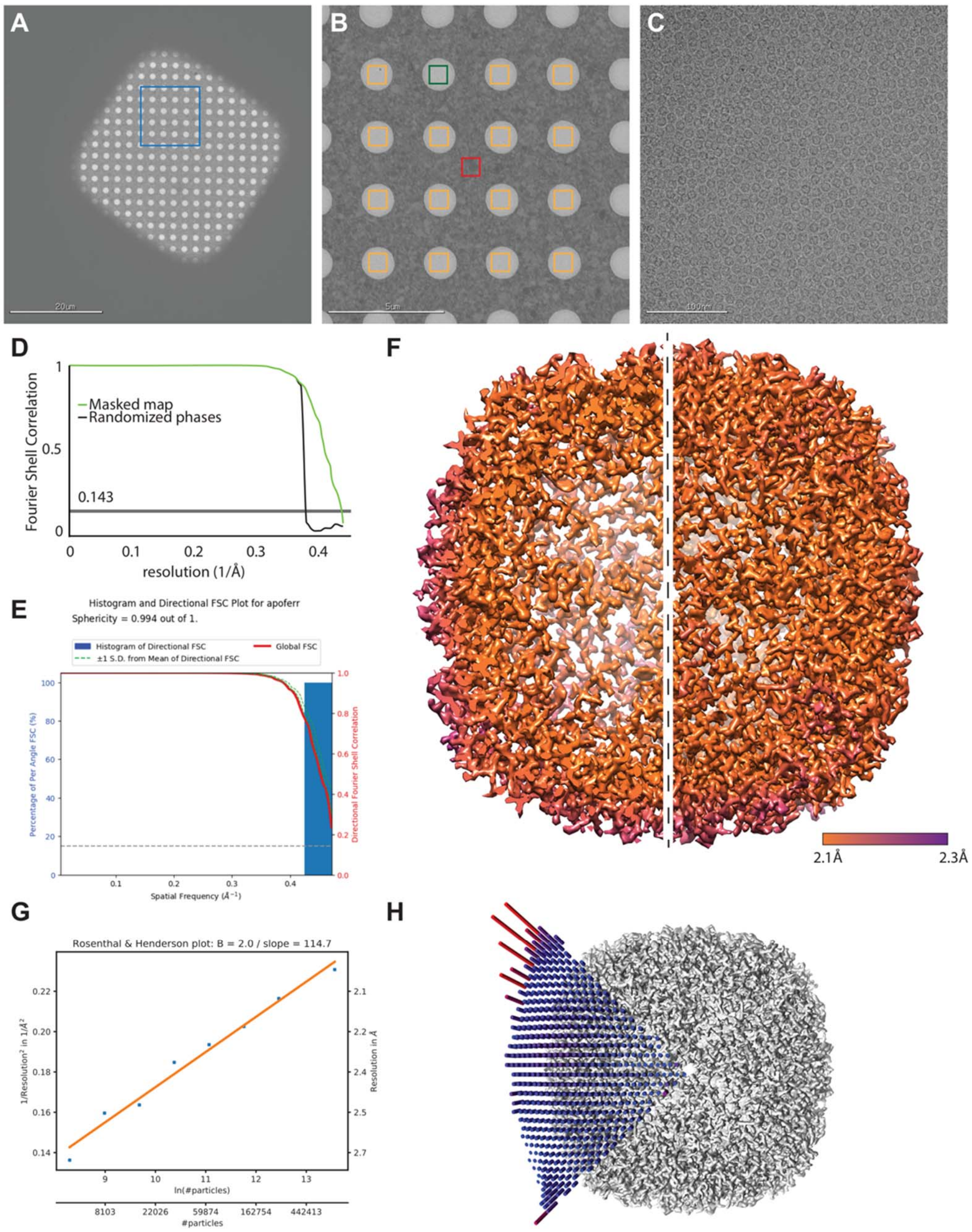

Figure S8 Exemplar micrographs and additional final reconstruction metrics for apoferritin imaged at 300KeV in 20% glycerol buffer. A. Exemplar grid square, a blue frame marks where micrograph B was acquired. B. An exemplar “hole” magnification micrograph, with a red square showing where real focus estimation was done, a green square shows where micrograph C was taken. C. An exemplar micrograph used for single particle picking and analysis. D. Fourier shell correlation curves of the masked map and the map with randomized phases, the grey line indicates the gold standard threshold. E. Directional FSC curves (as described in Supplementary Fig. 6E) showing how resolution varies by orientation (Tan *et al.*, 2017). F. Reconstruction volume colored by local resolution. G. B-factor plot showing how reconstruction resolution improves as larger subsets of the final particle set are used for 3D auto-refinement (Rosenthal &

Henderson, 2003), generated using the bfactor_plot.py script provided together with RELION3.1 source code. H. Angular distribution of views for the final reconstruction done assuming O symmetry.

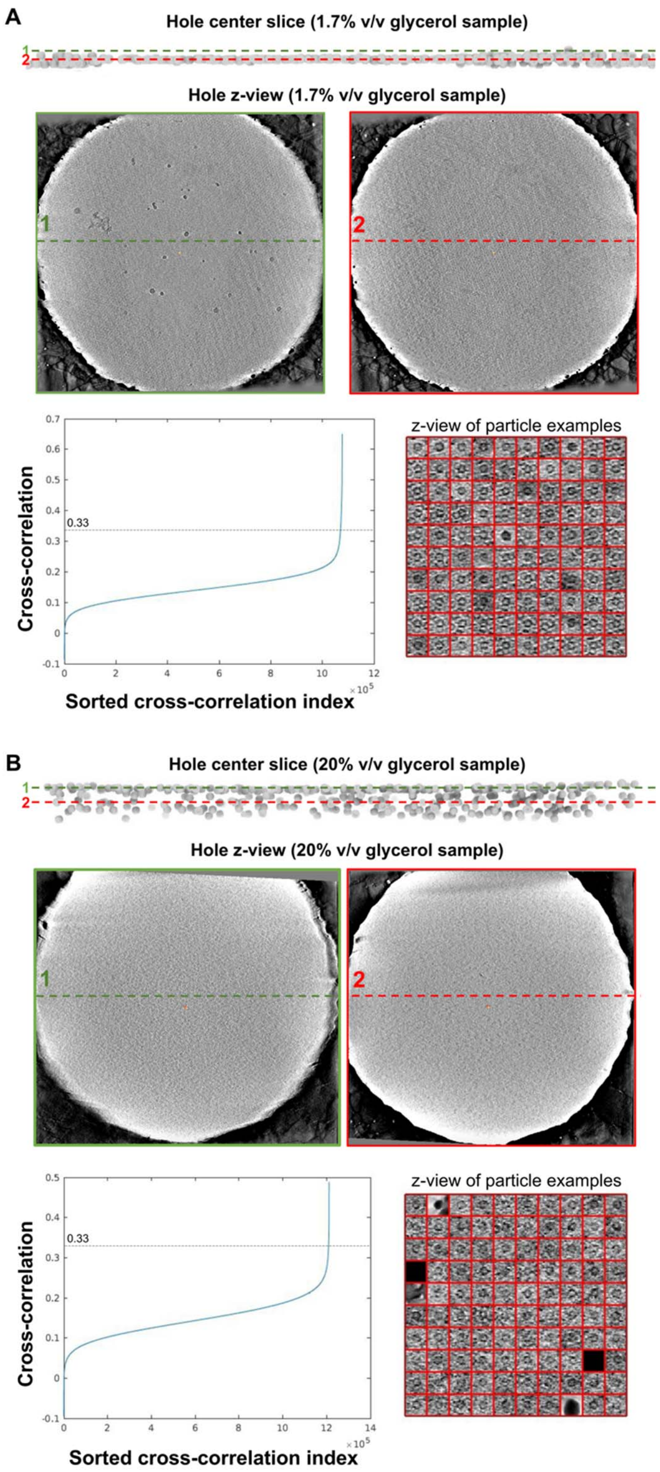

Figure S9 Distribution of particle layers in apoferritin samples with high and low glycerol concentrations. A. An exemplar hole from the 1.7% glycerol sample, showing two parallel planes in the aligned tomogram at different ice depths (center) labeled on a schematic profile (top) derived from template matching using an apoferritin model. The bottom left panel shows cross-correlation values as a function of peak index sorted by cross-correlation, the labeled line at 0.33 cross-

correlation indicates the threshold above which correlation peaks were used to construct the particle distribution model on the top. The bottom right panel shows 100 randomly-picked particles from the set used to construct the particle distribution model on the top, indicating the overwhelming majority of picks are clearly apoferritin particles. B. Same as A, but for a 20% glycerol sample.

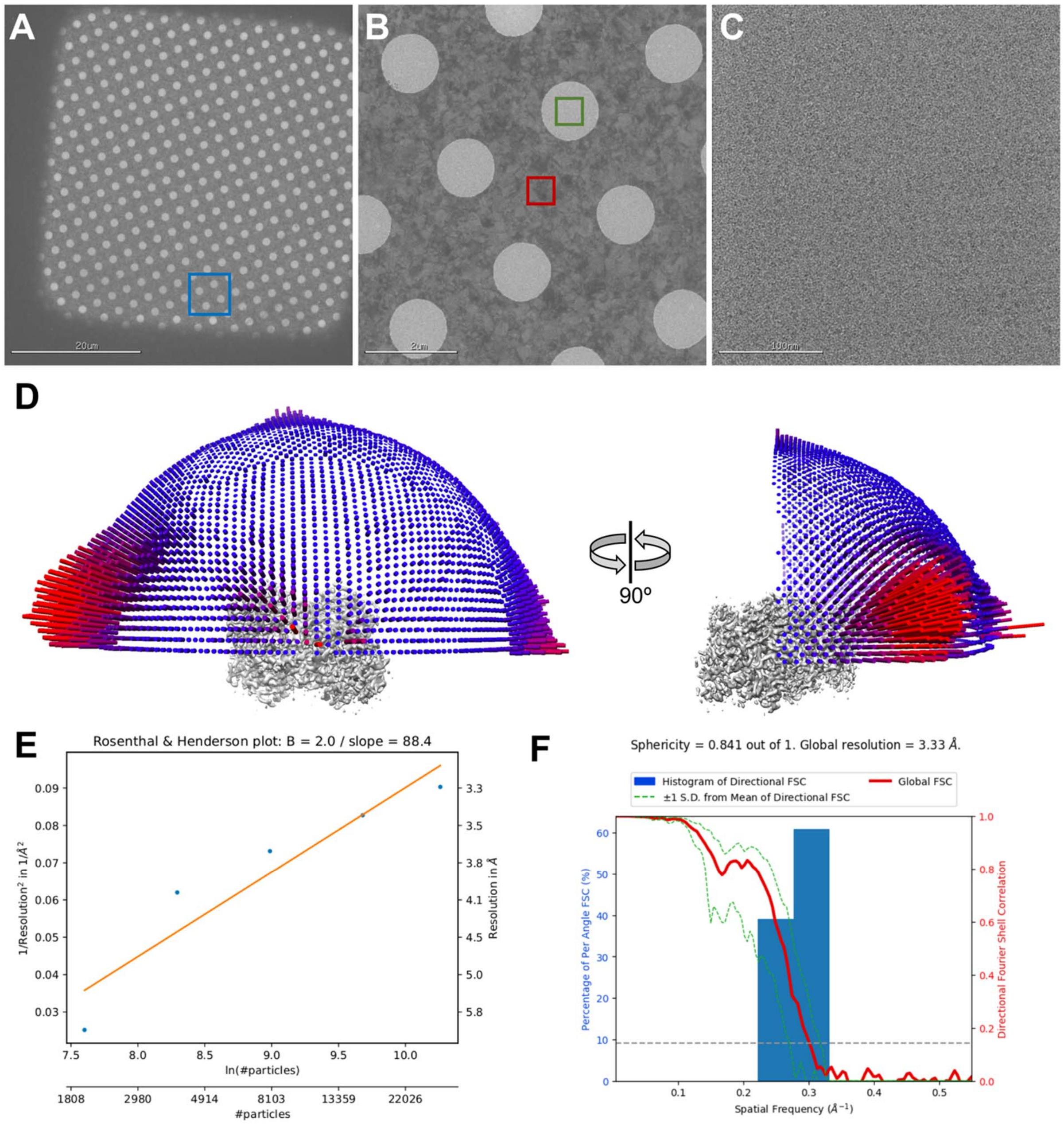

Figure S10 Exemplar micrographs and additional final reconstruction metrics for aldolase imaged at 200KeV in 20% glycerol buffer. A. Exemplar grid square, a blue frame marks where micrograph B was acquired. B. An exemplar "hole" magnification micrograph, with a red square showing where real focus estimation was done, a green square shows where micrograph C was taken. C. An exemplar micrograph used for single particle picking and analysis. D. Angular distribution of views for the final reconstruction done assuming D2 symmetry. E. B-factor plot showing how reconstruction resolution improves as larger subsets of the final particle set are used for 3D auto-refinement (Rosenthal & Henderson, 2003) , generated using the bfactor_plot.py script provided together with RELION3.1 source code. F.

Directional FSC curves (as described in Supplementary Fig. 6E) showing how resolution varies by orientation (Tan *et al.*, 2017).

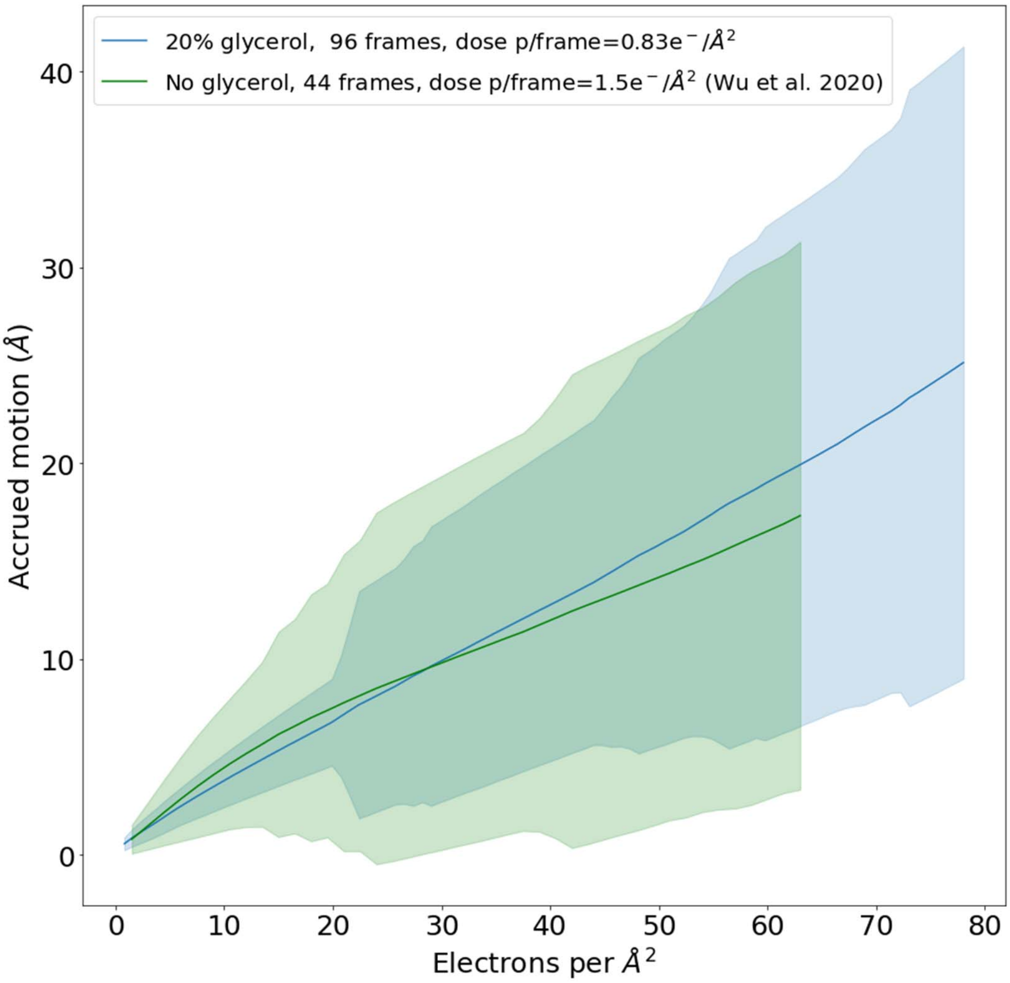

Figure S11 Accrued beam-induced motion for aldolase imaged at 200KeV in 20% glycerol buffer compared to a previous publication. Accrued beam-induced motion (per-frame summation) as a function of the number of electrons per Å^2 received by the sample. Our aldolase sample in 20% glycerol buffer, imaged at 200KeV (blue), compared to a previously published aldolase dataset without glycerol, with a similar imaging setup at 200KeV – green (Wu *et al.*, 2020). Solid lines: average over all collected movies at a given dose. Colored shades: Standard deviation over all movies at a given dose.

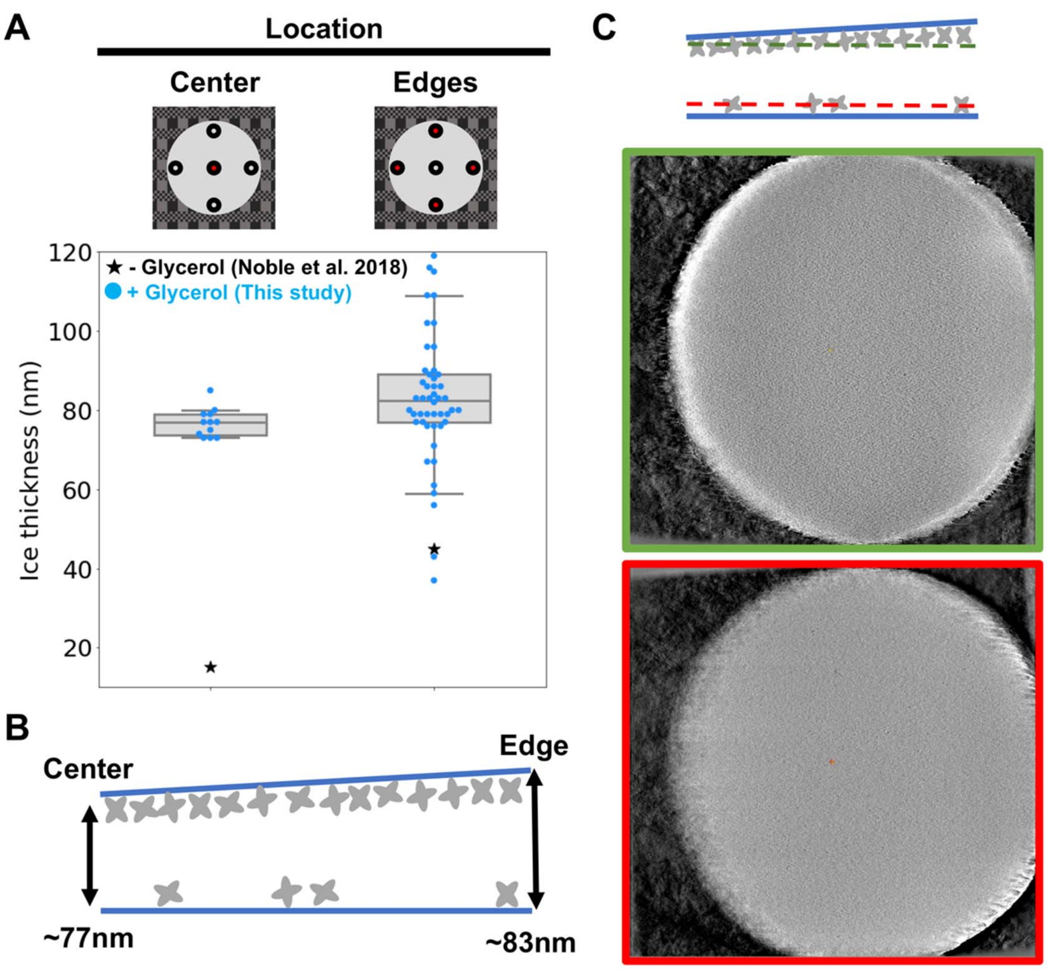

Figure S12 Ice thickness measurements obtained by electron tomography on an aldolase sample in 20% glycerol buffer. A. The hole diagrams on the top show the approximate locations of measurements in red, done either in the center or four edge spots. Data points are shown as colored dots (horizontally stacked only to aid clarity) overlaid on a grey box that spans the two central quartiles and is crossed by a vertical line at the median. The whiskers span the range of data between the 5th and 95th percentiles. Black stars represent data from previous studies for samples without glycerol (Noble *et al.*, 2018). B. Diagram of an average ice cross section for aldolase samples with 20% glycerol integrating all gathered information. The labeled values are the average ice thickness at the edges and center. C. Tomogram slices at the height of each of the two particle layers, with border colored according to the lines depicted above in the ice section cartoon.

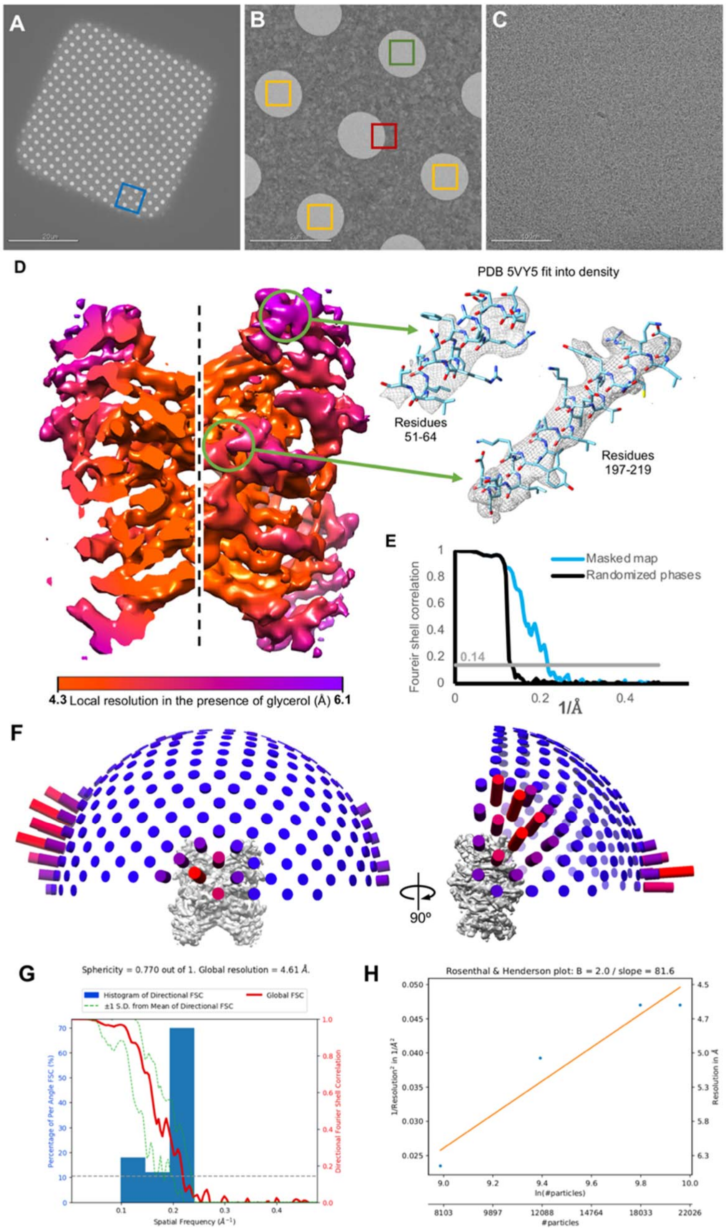

Figure S13: Exemplar micrographs and additional final reconstruction metrics for aldolase imaged at 300KeV in 20% glycerol buffer. A. Exemplar grid square, a blue frame marks where micrograph B was acquired. B. An exemplar “hole” magnification micrograph, with a red square showing where real focus estimation was done, a green square shows where micrograph C was taken. C. An exemplar micrograph used for single particle picking and analysis. D. Reconstruction volume colored by local resolution, as calculates by RELION3.1 (Zivanov *et al.*, 2018). Arrows point at two helices from areas with different overall resolution, with the 5VY5 PDB model fit into the density and displayed inside it. E. Fourier shell correlation curves of the masked map and the map with randomized phases, the grey line indicates the gold standard threshold. F. Angular distribution of views for the final reconstruction done assuming D2 symmetry. G. Directional FSC curves (as described in Supplementary Fig. 6E) showing how resolution varies by orientation (Tan *et al.*, 2017). H. B-factor plot showing how reconstruction resolution improves as larger subsets of the final particle set are used for 3D auto-

refinement (Rosenthal & Henderson, 2003) , generated using the bfactor_plot.py script provided together with RELION3.1 source code.

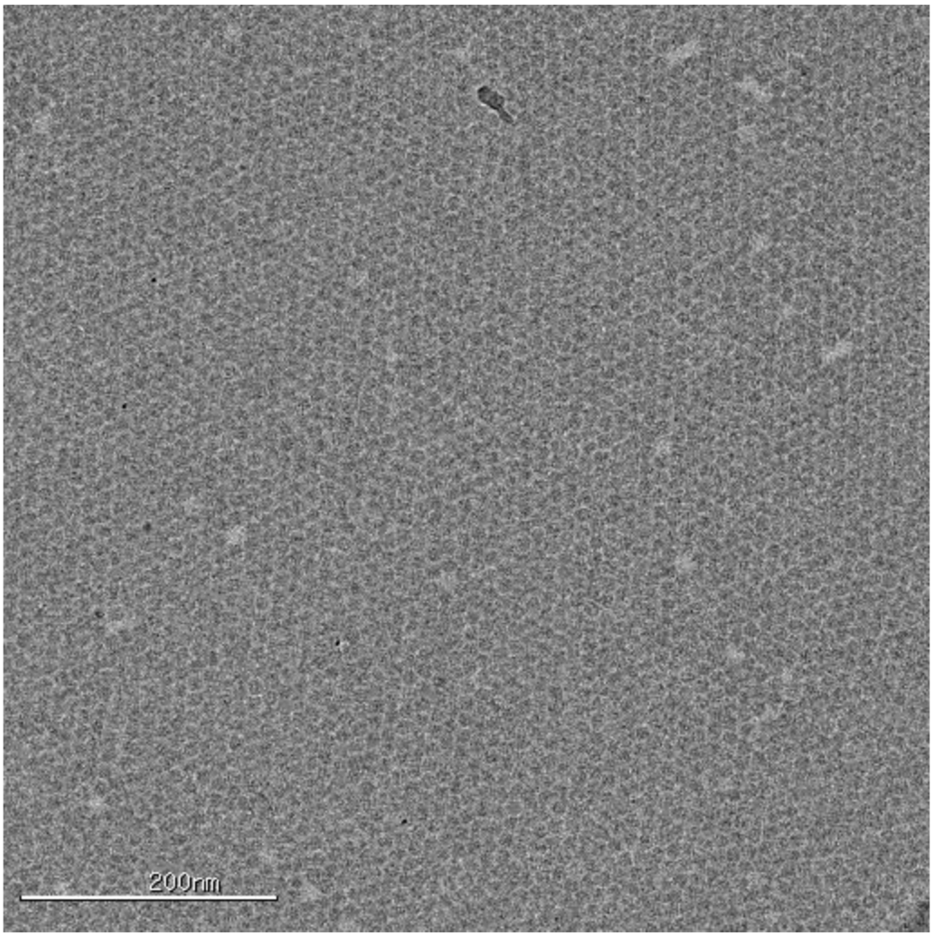

Figure S14 Exemplar micrograph obtained by plunging directly in liquid nitrogen. A clear array of particles, as usually formed in high-concentration samples of apoferritin, is visible in this image taken from a vitrified sample flash frozen in liquid nitrogen after side blotting, as done for negative stain. The pixel size is 1.77Å, and defocus -2.5μm.

Table S1 CryoEM data collection and processing parameters and statistics for mouse heavy chain apoferritin in 1.7% glycerol (200keV).

	Mouse heavy chain apoferritin in 1.7% glycerol (200keV)<br>**EMDB:** EMD-24795<br>**EMPIAR: EMPIAR-10857**
**Data collection**	
Microscope	Talos Arctica TEM (Thermo Fisher Scientific)
Voltage (keV)	200
Detector	Gatan K2 Summit (4k x 4k)
Magnification (nominal/calibrated)	36,000X / 43,478X
Exposure navigation	Image shift to 16 holes
Data acquisition software	Leginon (Suloway *et al.*, 2005)
Total electron exposure (e^-/Å^2)	49.82
Exposure rate (e^-/pixel/sec)	5.99
Frame length (ms)	100
Number of frames per micrograph	110
Pixel size (Å)	1.15
Defocus range (µm)	-0.4 to -4.2
Micrographs collected (no.)	1707
**Reconstruction**	
Image processing package	RELION 3.1
Total extracted particles (no.)	876,503
Refined particles (no.)	875,694
Final particles (no.)	598,660
Symmetry imposed	O
Resolution (Å)	
FSC 0.5 (unmasked / masked)	2.5Å / 2.4Å
FSC 0.143 (unmasked / masked)	2.4Å / 2.3Å
Resolution range (local)	2.3Å to 2.4Å
3DFSC Sphericity	0.997
Accuracy of translations / rotations	0.2Å / 0.24º
Sharpening B-factor (Å^2)	-19

Table S2 CryoEM data collection and processing parameters and statistics for mouse heavy chain apoferritin in 20% glycerol (200keV).

Mouse heavy chain apoferritin in 20% glycerol (200keV)<br>**EMDB:** EMD-24796<br>**EMPIAR: EMPIAR-10849**	
**Data collection**	
Microscope	Talos Arctica TEM (Thermo Fisher Scientific)
Voltage (keV)	200
Detector	Gatan K2 Summit (4k x 4k)
Magnification (nominal/calibrated)	36,000X / 43,478X
Exposure navigation	Image shift to 16 holes
Data acquisition software	Leginon (Suloway *et al.*, 2005)
Total electron exposure ($e^-/Å^2$)	50
Exposure rate (e^-/pixel/sec)	5.8
Frame length (ms)	100
Number of frames per micrograph	110
Pixel size (Å)	1.15
Defocus range (µm)	-0.3 to -4.0
Micrographs collected (no.)	2254
**Reconstruction**	
Image processing package	RELION 3.1
Total extracted particles (no.)	830,042
Refined particles (no.)	829,923
Final particles (no.)	683,154
Symmetry imposed	O
Resolution (Å)	
FSC 0.5 (unmasked / masked)	2.5Å / 2.4Å
FSC 0.143 (unmasked / masked)	2.3Å / 2.3Å
Resolution range (local)	2.3Å to 2.4Å
3DFSC Sphericity	0.993
Accuracy of translations / rotations	0.23Å / 0.37°
Sharpening B-factor ($Å^2$)	-36.12

Table S3 CryoEM data collection and processing parameters and statistics for mouse heavy chain apoferritin in 20% glycerol (300keV).

Mouse heavy chain apoferritin in 20% glycerol (300keV)<br>**EMDB:** EMD-24797<br>**EMPIAR: EMPIAR-10861**	
**Data collection**	
Microscope	FEI TITAN KRIOS
Voltage (keV)	300
Detector	Gatan K2 Summit (4k x 4k)
Magnification (nominal/calibrated)	29,000X / 48,732X
Exposure navigation	Image shift to 16 holes
Data acquisition software	Leginon (Suloway *et al.*, 2005)
Total electron exposure ($e^-/Å^2$)	50
Exposure rate (e^-/pixel/sec)	8.1
Frame length (ms)	100
Number of frames per micrograph	65
Pixel size (Å)	1.026
Defocus range (µm)	-0.8 to -1.5
Micrographs collected (no.)	1859
**Reconstruction**	
Image processing package	RELION 3.1
Total extracted particles (no.)	1,757,706
Refined particles (no.)	778,762
Final particles (no.)	778,762
Symmetry imposed	O
Resolution (Å)	
FSC 0.5 (unmasked / masked)	2.3Å / 2.2Å
FSC 0.143 (unmasked / masked)	2.1Å / 2.1Å
Resolution range (local)	2.1Å to 2.3Å
3DFSC Sphericity	0.99
Accuracy of translations / rotations	0.28Å / 0.41°
Sharpening B-factor ($Å^2$)	-117.6

Table S4 . CryoEM data collection and processing parameters and statistics for rabbit muscle aldolase in 20% glycerol (200keV).

	Rabbit muscle aldolase in 20% glycerol (200keV)<br>**EMDB: EMD-24799**<br>**EMPIAR: EMPIAR-10866**
**Data collection**	
Microscope	Talos Arctica TEM (Thermo Fisher Scientific)
Voltage (keV)	200
Detector	Gatan K2 Summit (4k x 4k)
Magnification (nominal/calibrated)	45,000 X / 54,945 X
Exposure navigation	Image shift to 4 holes
Data acquisition software	Leginon (Suloway *et al.*, 2005)
Total electron exposure ($e^-/Å^2$)	80
Exposure rate (e^-/pixel/sec)	3.46
Frame length (ms)	200
Number of frames per micrograph	96
Pixel size (Å)	0.91
Defocus range (µm)	-0.4 to -3.0
Micrographs collected (no.)	741
**Reconstruction**	
Image processing package	RELION 3.1
Total extracted particles (no.)	323,571
Refined particles (no.)	142,300
Final particles (no.)	28,588
Symmetry imposed	D2
Resolution (Å)	
FSC 0.5 (unmasked / masked)	6.1Å / 3.8Å
FSC 0.143 (unmasked / masked)	3.8Å / 3.3Å
Resolution range (local)	3.3Å to 4.6Å
3DFSC Sphericity	0.84
Accuracy of translations / rotations	0.54Å / 1.41º
Sharpening B-factor ($Å^2$)	-56.6

Table S5 CryoEM data collection and processing parameters and statistics for rabbit muscle aldolase in 20% glycerol (300keV).

	Rabbit muscle aldolase in 20% glycerol (300keV)<br>**EMDB:** EMD-24799<br>**EMPIAR: EMPIAR-10867**
**Data collection**	
Microscope	FEI TITAN KRIOS
Voltage (keV)	300
Detector	Gatan K2 Summit (4k x 4k)
Magnification (nominal/calibrated)	130,000 X / 47,846 X
Exposure navigation	Image shift to 5 holes
Data acquisition software	Leginon (Suloway *et al.*, 2005)
Total electron exposure (e^-/Å^2)	52
Exposure rate (e^-/pixel/sec)	5.73
Frame length (ms)	200
Number of frames per micrograph	50
Pixel size (Å)	1.045
Defocus range (µm)	-1.0 to -2.8
Micrographs collected (no.)	2591
**Reconstruction**	
Image processing package	RELION 3.1
Total extracted particles (no.)	1,255,598
Refined particles (no.)	143,234
Final particles (no.)	21,155
Symmetry imposed	D2
Resolution (Å)	
FSC 0.5 (unmasked / masked)	7.2Å / 6.2Å
FSC 0.143 (unmasked / masked)	6.2Å / 4.6Å
Resolution range (local)	4.3Å to 6.1Å
3DFSC Sphericity	0.77
Accuracy of translations / rotations	2.02Å / 6.82º
Sharpening B-factor (Å^2)	-197.1

Noble, A. J., Dandey, V. P., Wei, H., Brasch, J., Chase, J., Acharya, P., Tan, Y. Z., Zhang, Z., Kim, L. Y. & Scapin, G. (2018). *Elife* **7**, e34257.

Rosenthal, P. B. & Henderson, R. (2003). *Journal of molecular biology* **333**, 721-745.

Suloway, C., Pulokas, J., Fellmann, D., Cheng, A., Guerra, F., Quispe, J., Stagg, S., Potter, C. S. & Carragher, B. (2005). *J Struct Biol* **151**, 41-60.

Tan, Y. Z., Baldwin, P. R., Davis, J. H., Williamson, J. R., Potter, C. S., Carragher, B. & Lyumkis, D. (2017). *Nature methods* **14**, 793-796.

Wu, M., Lander, G. C. & Herzik Jr, M. A. (2020). *Journal of Structural Biology: X* **4**, 100020.

Zivanov, J., Nakane, T., Forsberg, B. O., Kimanius, D., Hagen, W. J., Lindahl, E. & Scheres, S. H. (2018). *elife* **7**, e42166.
